# PTS1 Peroxisomal Import Pathway Plays Shared and Distinct Roles to PTS2 Pathway in Development and Pathogenicity of *Magnaporthe oryzae*

**DOI:** 10.1371/journal.pone.0055554

**Published:** 2013-02-06

**Authors:** Jiaoyu Wang, Zhen Zhang, Yanli Wang, Ling Li, Rongyao Chai, Xueqin Mao, Hua Jiang, Haiping Qiu, Xinfa Du, Fucheng Lin, Guochang Sun

**Affiliations:** 1 State Key Laboratory Breeding Base for Zhejiang Sustainable Pest and Disease Control, Institute of Plant Protection Microbiology, Zhejiang Academy of Agricultural Sciences, Hangzhou, China; 2 College of Agriculture and Biotechnology, Zhejiang University, Hangzhou, China; Nanjing Agricultural University, China

## Abstract

Peroxisomes participate in various important metabolisms and are required in pathogenicity of fungal plant pathogens. Peroxisomal matrix proteins are imported from cytoplasm into peroxisomes through peroxisomal targeting signal 1 (PTS1) or peroxisomal targeting signal 2 (PTS2) import pathway. *PEX5* and *PEX7* genes participate in the two pathways respectively. The involvement of *PEX7* mediated PTS2 import pathway in fungal pathogenicity has been documented, while that of PTS1 remains unclear. Through null mutant analysis of *MoPEX5*, the *PEX5* homolog in *Magnaporthe oryzae*, we report the crucial roles of PTS1 pathway in the development and host infection in the rice blast fungus, and compared with those of PTS2. We found that *MoPEX5* disruption specifically blocked the PTS1 pathway. Δ*mopex5* was unable to use lipids as sole carbon source and lost pathogenicity completely. Similar as Δ*mopex7*, Δ*mopex5* exhibited significant reduction in lipid utilization and mobilization, appressorial turgor genesis and H_2_O_2_ resistance. Additionally, Δ*mopex5* presented some distinct defects which were undetected in Δ*mopex7* in vegetative growth, conidial morphogenesis, appressorial morphogenesis and melanization. The results indicated that the PTS1 peroxisomal import pathway, in addition to PTS2, is required for fungal development and pathogenicity of the rice blast fungus, and also, as a main peroxisomal import pathway, played a more predominant role than PTS2.

## Introduction

Peroxisomes are single membrane-bound organelles present in almost all eukaryotes [1]. These organelles contain more than 50 different enzymes involved in various metabolisms, such as fatty acid β-oxidation, glyoxylate cycle and degradation of reactive oxygen species (ROS) [2], [3]. Some special biochemical reactions, such as penicillin biosynthesis in *Penicillium* species, methanol utilization in yeasts and photorespiration in plants, also rely on peroxisomes [3], [4], [5]. The significance of peroxisomes in humans was demonstrated by diseases due to peroxisomal biogenesis disorders (PBDs), such as the Zellweger syndrome, the neonatal adrenoleukodystrophy, and the infantile Refsum’s disease [6].

Peroxisomes do not have their own internal DNA molecules [7], [8]. Their matrix proteins and membrane proteins have to be encoded by nucleic genes, synthesized in cytoplasm and translocated to the organelle via post-translational transport [1]. The import machinery of the peroxisomal proteins consists of another group of proteins, named peroxins, which are encoded by *PEX* genes. To date, dozens of the *PEX* genes have been identified in different eukaryotes [8]. Mutations of these genes usually alter the size and number of peroxisome or misallocate the peroxisomal proteins [9], [10], [11]. To be recognized by the import machinery, the peroxisomal matrix proteins usually contain specific conserved motifs known as peroxisomal targeting signals (PTSs). PTSs fall into at least two categories, PTS1 and PTS2. PTS1 is conserved tripeptide SKL or its derivative (S/C/A-K/R/H-L) at C-terminal, which presents in most of the known peroxisomal matrix proteins [12]. PTS2 has a consensus (R/K)-(L/V/I)-X5-(H/Q)-(L/A) located mainly at the N-terminus of a small amount of peroxisomal matrix proteins [13]. *PEX5* and *PEX7* genes encode the receptors for PTS1 and PTS2 respectively, which bind the peroxisomal matrix proteins directly or indirectly via co-receptors [14], [15]. *PEX5* was found to have cross-talking to *PEX7* during PTS2 import in plants and mammals [15], [16], [17], [18], [19], [20]. In recent years, much progress was made on the study of peroxisomal biogenesis in filamentous fungi [21], [22], [23], [24]. And the involvement of peroxisomes in host invasion of plant pathogenic fungi was also demonstrated in several species [23], [25], [26], [27].

*Magnaporthe oryzae* is a well-known pathogenic fungus that causes rice blast, one of the most devastating rice diseases. To penetrate the host surface, the fungus differentiates a well-specialized cell structure, appressorium. The appressoria are equipped with melanized cell wall and highly concentrated glycerol to generate enormous turgor [28]. Relying on the turgor, the fungus punches the host cuticles, penetrates into the cells, and subsequently grows within the host tissues.

Stored lipids in conidia are one source for the glycerol accumulation and are indispensable for the appressorium mediated infection [29]. During the pre-penetration stage, the lipids were degraded rapidly and migrated from germinated conidia into appressoria. In fungi, peroxisomes are the main location for lipid degradation [3]. The involvement of peroxisomal metabolism in the pathogenicity of *M. oryzae* was demonstrated by studies of *PEX6* and *PEX7* genes [23], [25], [30]. Disruption of *PEX7* blocked the PTS2 import pathway in *M. oryzae* [30]. *pex7* null mutant exhibited peroxisome related defects such as reduced lipid degradation, and lost the ability to cause disease on rice [30]. These data fully demonstrated the requirement of PTS2 pathway in fungal pathogenicity. *PEX6* gene encodes an AAA ATPase required in the recycle of PTS receptors [31]. *pex6* null mutant of *M. oryzae* lost the pathogenicity on rice and barley completely [30]. And further, the disruption of *PEX6* seemed to make more damages to the fungus than that of *PEX7* in conidiation, appressorial morphogenesis and melanization.

*PEX6* was previously demonstrated to be required in both PTS1 and PTS2 pathways [32], the differences in the *pex6* and *pex7* null mutants therefore hinted a possible role of PTS1 pathway in fungal pathogenicity. However, the contributions of PTS1 pathway to fungal development and fungal pathogenicity remain unclear in any plant pathogenic fungus so far. To clarify the functions of PTS1 pathway, we characterized the *PEX5* gene in *M. oryzae* (assigned as *MoPEX5*) by targeted gene replacement and compared it with *MoPEX7*. Our data showed that *MoPEX5* participated in PTS1 pathway in *M. oryzae* and play multiple roles in vegetative growth, conidiation, appressorial morphogenesis, H_2_O_2_ resistance and pathogenicity. Further, the findings let us propose that PTS1, as a main peroxisomal import pathway, play a more predominant role than PTS2 to fungal development and pathogenicity in the rice blast fungus.

## Materials and Methods

### Strains, Growth Condition and Transformation

*M. oryzae* wild-type strain Guy11 [33], transformants and mutants were grown routinely on complete medium (CM) [34] using standard procedures [35]. The carbon starvation (CM-C) and nitrogen starvation media (CM-N) were prepared as described [34]. Lipid medium was made by adding 0.5% (v/v) olive oil or Tween 80 into CM-C [34]. Glucose and sodium acetate medium were CM-C supplied with 6 mM glucose or 50 mm sodium acetate, respectively. For liquid cultivation, the conidia in 1×10^4^/ml were shaken at 150 rpm in liquid CM at 28°C. The *Agrobacterium tumefaciens*-mediated transformation (AtMT) was performed as described [36]. CM plates containing corresponding antibiotics (250 µg/ml hygromycin B (Calbiochem, Germany), 200 µg/ml glufosinate-ammonium (Sigma, USA) or 800 µg/ml G418 (Sigma, USA) were used in screening transformants.

### cDNA Cloning of MoPEX5 and MoPEX7

The cDNA fragment of *MoPEX5* and *MoPEX7* were amplified from a cDNA library constructed with RNA of *M. oryzae* Guy11 [37] using primer-pairs p5cds3-Xb/P5cds4-Bm, and P7cds3-Xb/p7cds-Bm respectively. The amplicons were cloned into pGEM-T easy vector (Promega, USA) and sequenced by Invitrogen, USA. The coding information of the sequences was analyzed at the GENSCAN web server at MIT (http://genes.mit.edu/GENSCAN.html) and the protein phylogenies were analyzed by using MEGA 5.0 software.

### Construction of Gene Disruptants, Recovery Transformants and Fluorescent Transformants

Construction of the gene disruption vectors (p1301XGG-PEX5 and p1301XGG-PEX7), mutant screening and verification by PCR amplification and Southern blotting were described previously [32]. The selected mutant candidates were further confirmed by reverse transcription PCR using the primer pairs p5RTcheck1/p5RTcheck2 (for *MoPEX5*) and p7RTcheck1/p7RTcheck2 (for *MoPEX7*). One mutant strain of each gene, which showed typical phenotypes, was selected for mutant complementation. The 4.6-kb fragment containing full length *MoPEX5* coding region and 1.5-kb upstream was amplified using primer pair P5f-Xb/P5r-Sc and cloned into pCR-XL-TOPO vector (Invitrogen, USA) to generate pTOPO-pex5. The 3.5-kb PCR fragment containing *MoPEX7* coding region and 1.5-kb upstream was amplified using primer pair P7f-Xb/P7r-Ec and cloned into pCR-XL-TOPO to generate pTOPO-pex7. The *Xba*I-*Sac*I digested *MoPEX5* and *Xba*I-*Eco*RI digested *MoPEX7* fragments were inserted respectively into the corresponding sites in p1300BAR, a binary vector provided with glufosinate-ammonium resistance [32], to generate complementation vectors p1300BAR-*MoPEX5* and p1300BAR-*MoPEX7*. Then the two complementation vectors were introduced into corresponding mutants respectively via AtMT. The transformants were selected and verified by PCR and Southern blotting. Strains with single-copy integration were selected and analyzed. To monitor the gene expression, the 1.5 kb promoter regions of *MoPEX5* and *MoPEX7* were amplified by using the primer pairs P5Pf-Pv/P5Pr-Xb and P7Pf-Pv/P7Pr-Xb respectively. The amplificons were then inserted into pBMGFP [32], a vector containing glufosinate-ammonium resistance and GFP expression cassette under *MPG1* promoter, by *Pvu* I/*Xba*I digestion, to replace the promoter and generate pBP5GFP and pBP7GFP. The pBP5GFP and pBP7GFP were integrated into Guy11 and resulted transformants were observed microscopically. For fluorescent col-localization, the RFP fusion vector p1300NMRFPA which carried G418 resistance, and GFP fusion vector pBARMGFPB which carried glufosinate-ammonium resistance [32], were integrated together into the wild type or mutant strains. The transformants were selected via the corresponding antibiotic and confirmed by PCR and Southern blotting. Of them, the single-copy integrated transformants were selected for further observation. All the primers used in present study were list in Table 1.

**Table 1:** The primers used in this study.

Name	Sequence (5′-3′)	To amplify
p5cds3-Xb	GCTCTAGAATGTCGTTCATGGGAGGGGCCG	*MoPEX5* cDNA
P5cds4-Bm	CGGGATCCTAAAAGTCAAAGTCCTTCCTG	
P7cds3-Xb	GCTCTAGAATGAGTGCTCCTATGCTTGAGTTCC	*MoPEX7* cDNA
P7cds4-Bm	CGGGATCCATCGTGTAAGGCTGGTCAAGTCGTG	
P5f-Xb	TTTCTGTTGTCACGGTTCTAGAGG	Full length of *MoPEX5*
P5r-Sc	CACGGCATACCGAGCTCAGGGTGG	
P7f-Xb	GCTCTAGACTGGCTTCAGGCGGACATACAGAA	Full length of *MoPEX7*
P7r-Ec	TGTGATTTGTGAATTCTGTGCTCG	
P5Pf-Pv	CACATCTGGTTCATTGACTA	Promoter fragment of *MoPEX5*
P5Pr-Xb	ACAAGAACGTCGCCATGTCG	
P7Pf-Pv	GCTGGCTTCAGGCGGACATA	Promoter fragment of *MoPEX7*
P7Pr-Xb	AGCACTCATCATGAATAAGA	

* The restriction sites used were underlined.

### Nucleic Acid Manipulations and Southern Blot

Genomic DNAs were isolated by using the CTAB (hexadecyltrimethylammonium bromide) method [34] from 2-day-old mycelia in CM cultures shaken at 27°C, 150 rpm. Total RNA for cDNA amplification and reverse-transcription PCR was isolated by using Trizol reagent from mycelia treated in same way. Electrophoresis and restriction digestion were carried out using the standard procedures (Sambrook *et al*., 1989). Probe labeling and DNA hybridization were processed by using a digoxin labeling and detection kit with the procedure following the kit instruction (Roche, Germany).

### Plant Infection Assays

For spray inoculation, 2×10^4^ and 2×10^5^ conidia/ml suspensions supplied with 0.25% gelatin and the seedlings of 18-day-old rice CO39, 6-day-old barley ZJ-8 and 22-day-old *Brachypodium distachyon* Bd21 were used. In each treatment, 4 ml conidia suspension was sprayed on 15 seedlings in one pot. Three replicates were set for each treatment. Inoculated plants were incubated in a controlled chambers first at 24°C, 99% humidity and darkness for 24 h and then altered to 24°C, 84% humidity, with 900 E/m2/s tungsten illuminations for 12 h per day for 5–10 d. The experiment was replicated for 3 times. For detached leaf inoculation, 20 µl suspensions in 1×10^5^ conidia/ml were dropped on 5 cm leaf segments of 6-day-old seedlings of barley ZJ-8. The incubated leaf segments were put in Petri dishes where 3 layers of filter paper soaked with sterile water were tiled to keep humidity and placed in the same environment-controlled champers as above. For wounded leaf inoculation, the leaf segments were first slightly abraded with a mini file to remove the cuticle. For microscopic observation of host invasion, the infected barley leaf segments were discolored with methanol, fixed with 1 M KOH, heated at 70°C for 30 min, stained with aniline blue, then observed and photographed by epifluorescence microscopy as described [38].

### Pathogenicity Related Phenotypic Analysis

Five-mm diameter mycelia discs were incubated on 90 mm plates of CM, lipid medium, glucose medium at 28°C, 12-h day length to compare the vegetative growth. The colonial diameters were measured after 4 d and 10 d. The conidiogenesis was measured on CM plates after 8 d to 16 d. The conidia harvested from 8-day-old CM plates were incubated on hydrophobic membrane in 5×10^4^ conidia/ml and the germination rates after 2, 4, 8 and 24 h and appressoria formation frequencies after 4, 8 and 24 h were tested. The turgor of 24 h appressoria was measured using the cytorrhysis/plasmolysis test. Conidial harvest, appressorium induction and appressorial turgor measurement were performed as described previously [39].

### Fluorescent and Transmission Electron Microscopy

The fluorescence of FDA, Nile red and aniline blue were observed under fluorescent microscope Olympus Xa21 (Olympus, Japan). GFP or RFP fluorescence was observed using the Leica SP2 Confocal System (Leica, Germany). The objective was a 63×Plan-Apochromat (numerical aperture, 1.4) oil immersion lens. Images were taken though 488 nm wavelength laser excitation and 505–530 nm band pass emission filter. The hyphae and conidia harvested from 8-d-old CM plate and the appressoria incubated for 24 h were collected to perform TEM (transmission electronic microscopy). The electron microscope was JEM-1230 (JEOL, Tokyo, Japan). The samples treatment was processed as the procedure described [39].

### Nile Red, FDA and H_2_O_2_ Assays

Nile red staining for conidia and appressoria was performed as described [39]. The viability of conidia was tested by incubating 1×10^5^/ml conidia in 100 µg/ml FDA (fluorescein diacetate) solution for 3 min [40]. The viable conidia which could be stained and exhibited green fluorescence under a fluorescent microscope were counted and statistically analyzed. To assess the restraining of H_2_O_2_ to conidial germination, the 2×10^6^ conidia were spread on a 90-mm CM plate and a 5-mm medium discs containing 0.5 M H_2_O_2_ was put on the center. The plates were incubated in 28°C for 3 days and the restraining rings formed were measured.

## Results

### Isolation and Disruption of MoPEX5

**Figure 1:**
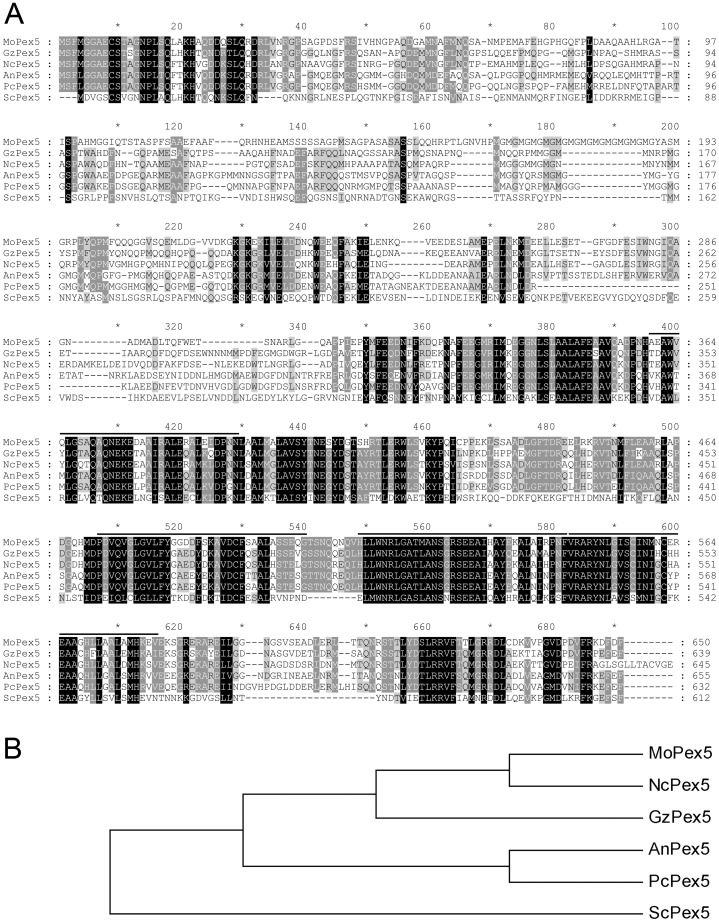
Predicted amino acid sequence and phylogenetic analysis of MoPex5. (A) Alignment of predicted amino acid sequence of MoPex5 and its homologues. The amino acid sequences of GzPex5 (EAA68640) from *Gibberella zeae*, NcPex5 (EAA36111) from *Neurospora crassa*, AnPex5 (CBF85028) from *Aspergillus nidulans*, PcPex5 (AY366189) from *Penicillium chrysogenum* and ScPex5 (CAA89730) from *Saccharomyces cerevisiae* were aligned with Clustal W. Identical amino acids are highlighted against a black background, conserved residues are shown on a dark gray background, and similar amino acids are shown on a light gray background. Four probable TPR domains of MoPex5 were indicated with a line on the top of the sequence. (B) Phylogenetic relationship among MoPex5 and its homologues. The phylogenetic trees of the amino acid sequences were created using the MEGA 5.0 program according to the result of alignment.

The putative homologues of *PEX5* (MGG_10840) was identified by searching the Pex5p (CAA89730) of *S. cerevisiae* against *M. oryzae* genome using the blastP procedure [41], and assigned as *MoPEX5*. The cDNA amplicon showed that the open reading frame (ORF) of *MoPEX5* was 2,044-bp long, contained 1 intron, and encoded a 650-aa peptide (MoPex5) which exhibited 57% aa identity to peroxin 5 of *N. crassa* (EAA36111) and 33% to that of *S. cerevisiae* (AAA64794) and contained four TPR domains (Figure 1A&B).

Previously, we deleted *MoPEX5* and *MoPEX7* respectively in the wild type strain Guy11 through *A. tumefaciens*-mediated transformation [42]. To determine the roles of *MoPEX5*, the Δ*mopex5* mutants were phenotypically analyzed. In view of the Δ*mopex7* mutant described from strain KJ201 [30], the Δ*mopex7* mutants from Guy11 were parallelly analyzed as a comparison with Δ*mopex5*.

### MoPEX5 Disruption Blocked PTS1 Peroxisomal Import Pathway

**Figure 2:**
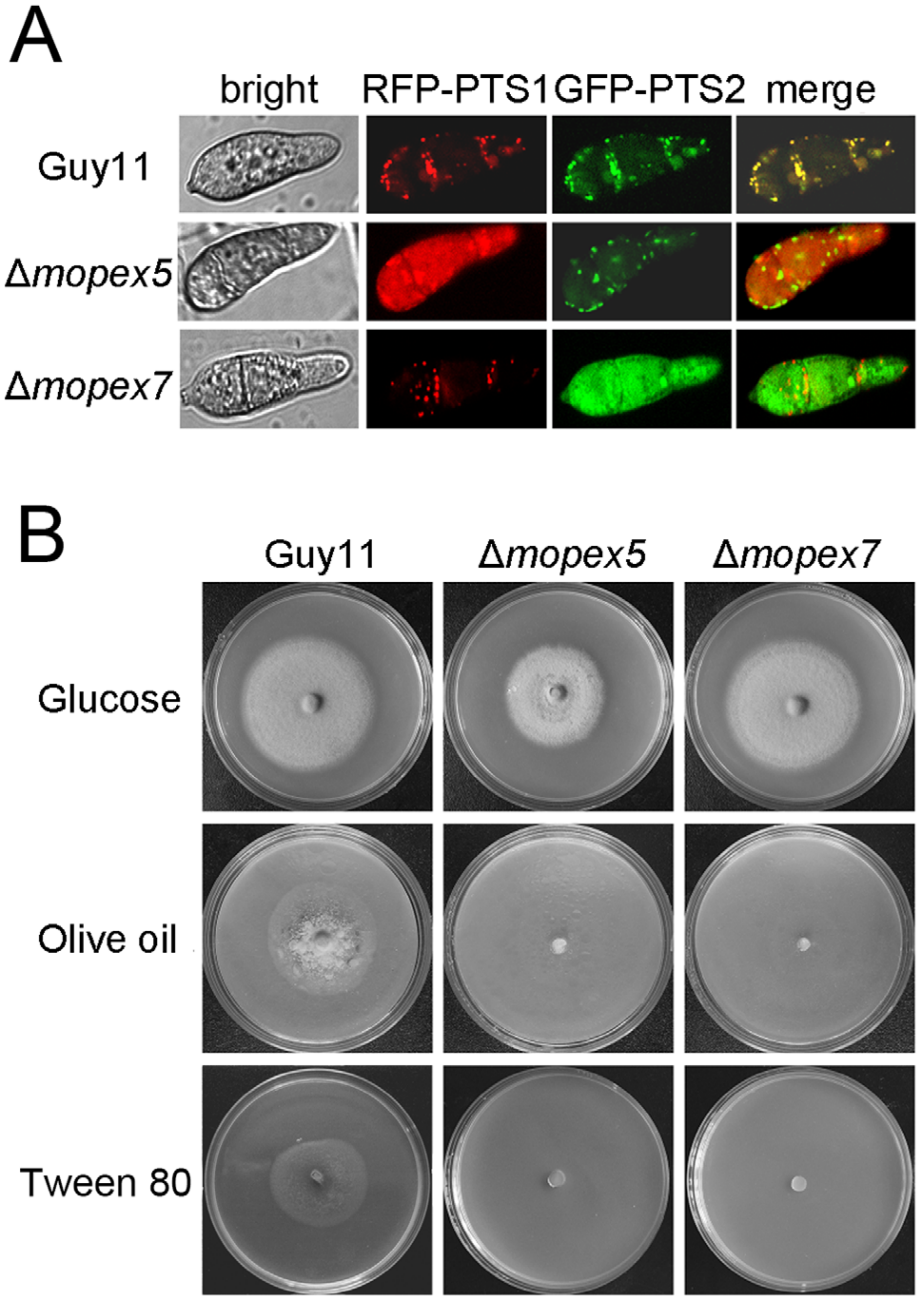
Mutation of *MoPEX5* blocked the PTS1 peroxisomal import pathways and affected the lipid utilization. (A) The peroxisomal localization of the RFP-PTS1 and GFP-PTS2 in the wild type, Δ*mopex5* and Δ*mopex7*. Conidia of the transformants Guy11/RFP-PTS1::GFP-PTS2, Δ*mopex5*/RFP-PTS1::GFP-PTS2 and Δ*mopex7*/RFP-PTS1::GFP-PTS2 harvested from 8-day-old CM plates were observed under a confocal fluorescence microscopy. (B) Lipid utilization of the wild type, Δ*mopex5* and Δ*mopex7*. The Strains were cultured on medium with glucose, olive oil or Tween 80 as sole carbon source for 12 d at 28°C.

To investigate the functions of *MoPEX5* in peroxisome biogenesis, the sorting of fluorescent proteins fused with PTS1 or PTS2 in Δ*mopex5* were tested. The RFP fused with a C-terminal consensus motif SRL [43] (RFP-PTS1) and GFP with an N-terminal PTS2 (GFP-PTS2) were co-introduced into Δ*mopex5*, Δ*mopex7* and the wild-type respectively. The verified transformants were observed by a laser confocal microscope. In the transformants derived from the wild type strain, both the red and green fluorescence were detected predominantly as puncta at the peripheral region of the cells, indicating the proper peroxisomal localization of both PTS1 and PTS2 proteins (Figure 2A). In the transformants from the Δ*mopex5* strain, fluorescence of RFP-PTS1 was dispersed in cytoplasm while that of GFP-PTS2 remained punctate. In contrast, in the transformants from the Δ*mopex7* strain whose PTS2 pathway was blocked, RFP-PTS1 was seen punctate while GFP-PTS2 was cytoplasmic. Together with the related findings in yeasts and other fungal species [21], [24], [30], the result indicated that *MoPEX5* gene, in contrast to *MoPEX7*, was involved PTS1 but not PTS2 peroxisomal import pathway.

Due to the peroxisomal defects, lipid utilizing in the Δ*mopex5* was negatively affected (Figure 2B). On medium with lipids (olive oil or Tween 80) as soul carbon source, the growth of Δ*mopex5* was abolished, in contrast to the efficient growth of the wild type strain. On glucose medium, Δ*mopex5* grew normally, indicating its glucose utilization was unaffected. Further, Δ*mopex5* remained the ability to use sodium acetate as carbon source (Figure S1). These data indicated that *MoPEX5* was required in lipids metabolism in *M. oryzae*. And meanwhile, Δ*mopex7* mutant was also deficient in lipids utilization.

### MoPEX5 was Essential for Host Infection

**Figure 3:**
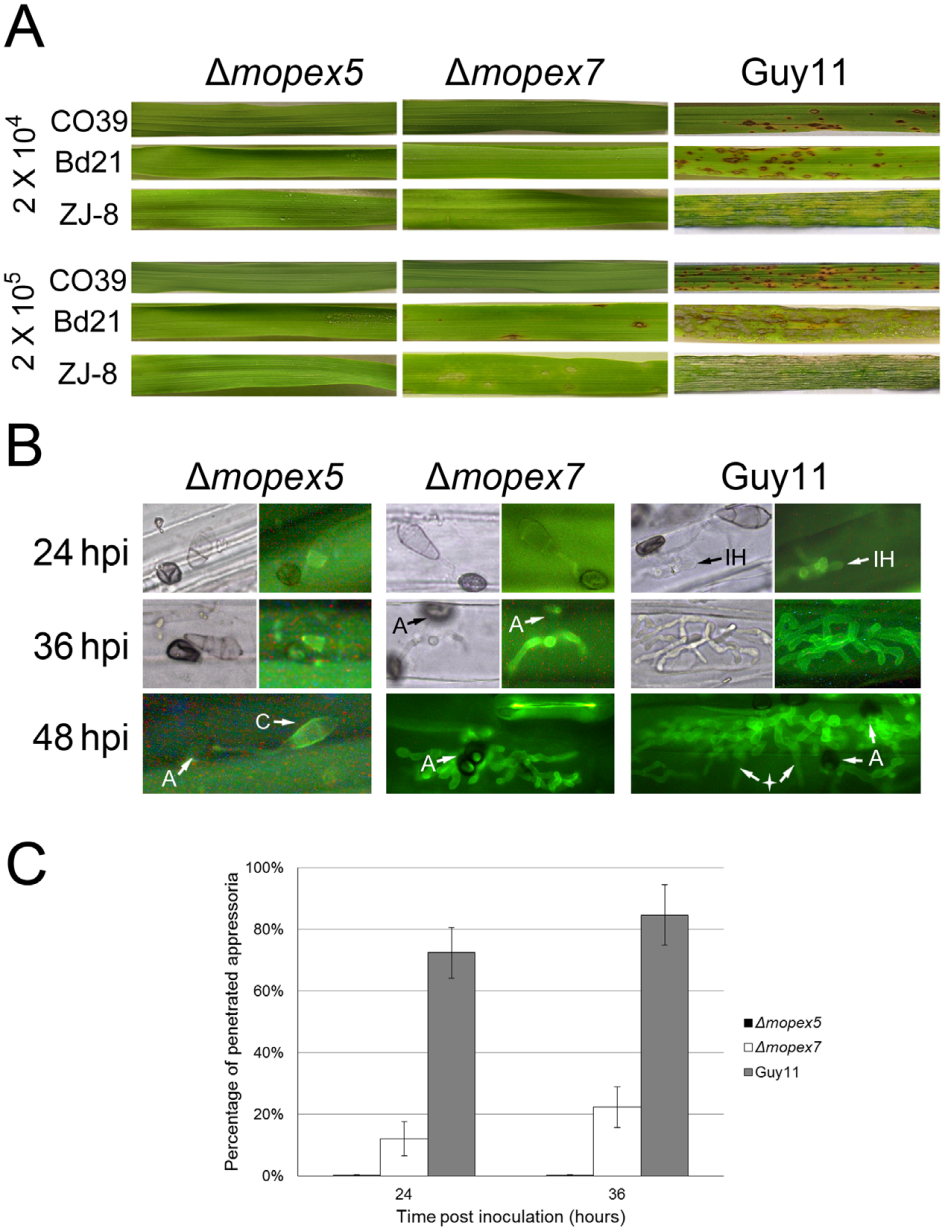
Host infection of the wild type, Δ*mopex5* and Δ*mopex7*. (A) The symptoms on the rice cultivar CO39, *Brachypodium distachyon* Bd21 and Barley ZJ-8 after spray inoculation with conidial suspension in 2×10^4^ and 2×10^5^ conidia/ml and the for 5 d. (B) The inoculated barley leaves were discolored, strained with aniline blue and observed under a fluorescent microscope. C, conidia; A, appressoria; IH, infection hyphae. The four-pointed star indicated hyphae that passed through the cell wall and invaded the neighbor cell. (C) The frequencies of penetration pegs formed by the appressoria after 24 h and 36 h on barley leaves were statistically compared.

To investigate the roles of *MoPEX5* in plant disease, we performed pathogenicity test on rice, barley, and *Brachypodium distachyon* with two conidial suspensions (2×10^4^ and 2×10^5^ conidia/ml). At 4 days post inoculation (dpi), the leaves inoculated with the wild type conidia in either concentration produced large numbers of typical lesions that developed rapidly (Figure 3A). In contrast, Δ*mopex5* at either conidia concentration generated no lesions on any host, indicating its pathogenicity was lost completely. As described, Δ*mopex7* didn’t lead to symptoms on rice [30]. On barley and *B. distachyon*, at the lower conidial concentration, Δ*mopex7* was also nonpathogenic. But when the conidial concentration was improved, typical lesions, although much less than those of the wild type, were generated on Δ*mopex7* inoculated leaves of barley and *B. distachyon*. These results indicated that *MoPEX5*, as well as *MoPEX7*, was required to the pathogenicity of *M. oryzae*. And *MoPEX5* was likely more contributional than *MoPEX7*.

The histological evidence of the pathogenic defects was found by microscopic observation of the infectional development on barley leaves (Figure 3B&C). At 24 h post inoculation (hpi), most of the wild-type appressoria (72.4%) had penetrated into the host cells and formed branched or still unbranched infectious hyphae. The infectious hyphae expanded rapidly in the infected cells in subsequent 6∼10 hours and filled them up to 36 hpi. At 48 hpi, the infectious hyphae extruded their tips out of the first infected host cells and invaded the neighbor ones. The penetration ability of Δ*mopex5* was lost completely. No efficient penetration was found on the leaves inoculated with Δ*mopex5*. The penetration ability of Δ*mopex7* reduced remarkably. Most of its appressoria (88.3%) kept un-penetrated up to 24 hpi, and the typical infectious hyphae could not be found until 36 hpi. Meanwhile, the growth of infectious hyphae of Δ*mopex7* was slowed down, which took about 12 h or more to fill the first infected cells. These data, corresponding with the result of pathogenicity test, indicated that the *MoPEX5*, as well as *MoPEX7*, was required in host infection, and this requirement likely related to both cuticle penetration and further development within host cells.

### MoPEX5 Disruption Resulted in Reduced Vegetative Growth and Abnormal Colonial Morphology

**Figure 4:**
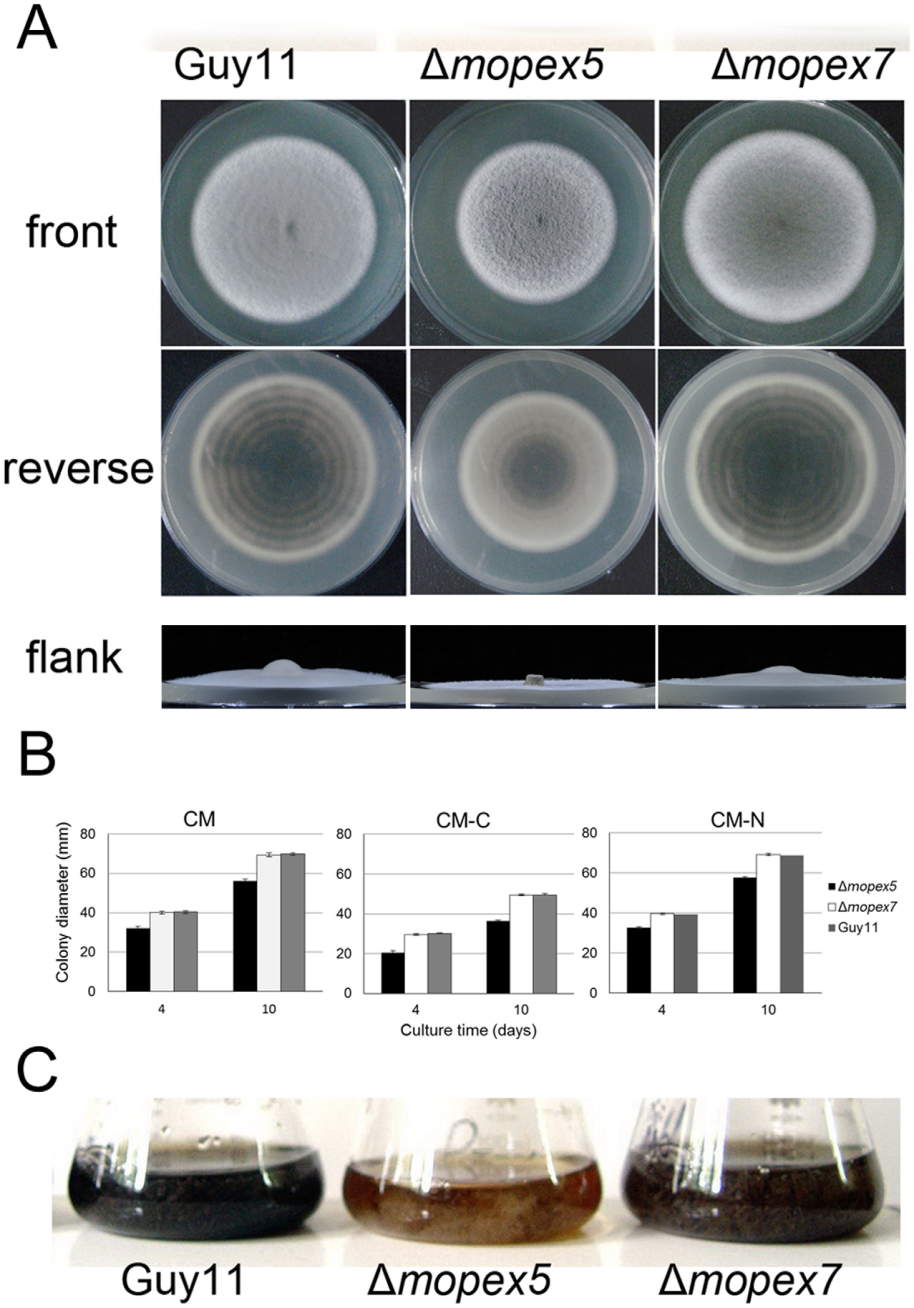
Vegetative growth and mycelial melanization of the wild type, Δ*mopex5* and Δ*mopex7*. (A) The colonies of the strains cultured on the complete media (CM) plates for 10 d. (B) Colonial diameters of the strains were measured and statistical analyzed after cultured on CM, CM-C and CM-N for 4 d and 10 d. (C) The conidia in 1×10^4^/ml of the strains were cultured in liquid CM shaking at 150 rpm at 28°C with 24 h day length, then the colors of the cultures were observed after 5 d.

To investigate the reasons for the penetration defects of Δ*mopex5*, possible pathogenicity-related phenotypes were analyzed. On complete media (CM), Δ*mopex5* grew slower and formed sparse and fascicular aerial hyphae, in contrast to the wild type and Δ*mopex7* (Figure 4A). On carbon or nitrogen starvation media, Δ*mopex5* also exhibited reduced growth compared with the wild type and Δ*mopex7* (Figure 4B). The melanization of Δ*mopex5* colony was reduced on CM. This reduction was also found in liquid cultures. In liquid CM, the wild type and Δ*mopex7* produced abundant melanin which made their cultures dark, while Δ*mopex5* in same conditions exhibited much lighter color (Figure 4C). This result indicated that *MoPEX5*, unlike *MoPEX7*, was involved in vegetative growth and colonial morphology of *M. oryzae*.

### MoPEX5 Disruption Reduced Conidial Genesis and Viability

**Figure 5:**
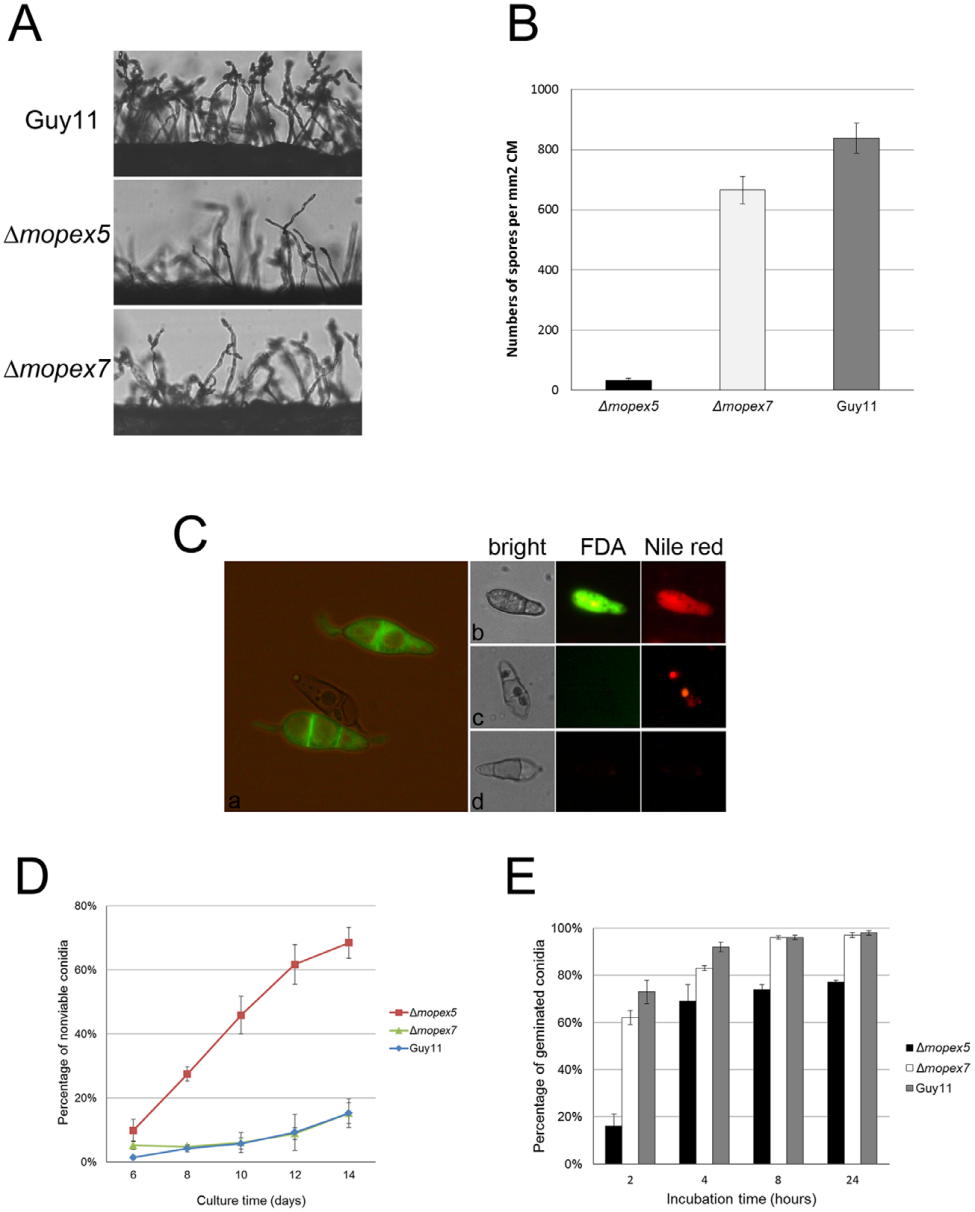
Conidial generation and viability of Δ*mopex5*, Δ*mopex7* and the wild type. (A) The development of the conidia and conidiophores of the strains grown on complete medium. (B) Statistical analysis of conidial production. The conidia produced by the strains grown on complete medium for 9 days were collected and counted. (C) Fluorescence staining of the conidia of the strains. The conidia were strained with fluorescein diacetate (FDA) and Nile red then observed under a fluorescent microscope. a, the viable conidia stained with FDA emitted bright green fluorescence while the dead ones did not; b, a viable conidium stained with FDA and Nile red; c and d, nonviable conidia strained with FDA and Nile red. (D) Percentages of nonviable conidia of the strains on complete media cultured for different time (E) Germination rates of conidia of the strains harvested from 8-day- old complete medium and incubated on inducible membrane.

The generation of conidia and conidiophores of Δ*mopex5* strain reduced dramatically (Figure 5A). On 9-day-old CM plates, Δ*mopex5* produced 32.1±6.21 conidia per mm^2^, equivalent to only 3.8% of that of the wild type (Figure 5B). Δ*mopex7* produced 665.3±45.5 conidia per mm^2^, reducing to 79.3% of the wild type. Moreover, the conidia of Δ*mopex5* were nonviable in much higher proportion than those of the wild type and Δ*mopex7*. The nonviable conidia were easily distinguished by their incapability of FDA staining (Figure 5C). These nonviable conidia were either hollow with only shells or with granular residues inside their cells. The residues were capable of Nile red staining, indicating that their main components were lipids. Additionally, the proportion of the nonviable conidia of Δ*mopex5* increased rapidly along the culture time (Figure 5D). On 6-day-old CM plates, 10 percent of Δ*mopex5* conidia were nonviable, slightly higher than those of the wild type and Δ*mopex7*. Whereas on 14-day-old CM plates, the percentage of nonviable conidia of Δ*mopex5* reached to 70%, in contrast to those of Δ*mopex7* and the wild type which were still under 20%. The lower viability of spores was also found in *pex5* mutant of *Fusarium graminearum*, which was related to the accumulation of ROS followed by necrotic cell death [44].

The less viability of Δ*mopex5* conidia was resulted possibly from the defective lipids conversion. The conidial viability was also reflected in their germination. Harvested from 8-day-old CM plates and incubated on an inductive membrane for 24 h, 98% conidia of the wild type geminated, while only 77% of Δ*mopex5* did (Figure 5E), corresponding with the result (76%) in FDA assay. Δ*mopex7* exhibited a slight delay in germination at first 4 h but no final difference to the wild type at 24 h. We considered that the low conidial viability and the reduced germination was the first reason for the failure of Δ*mopex5* in infectivity.

### MoPEX5 was Required for Appressorial Formation and Morphology

**Figure 6:**
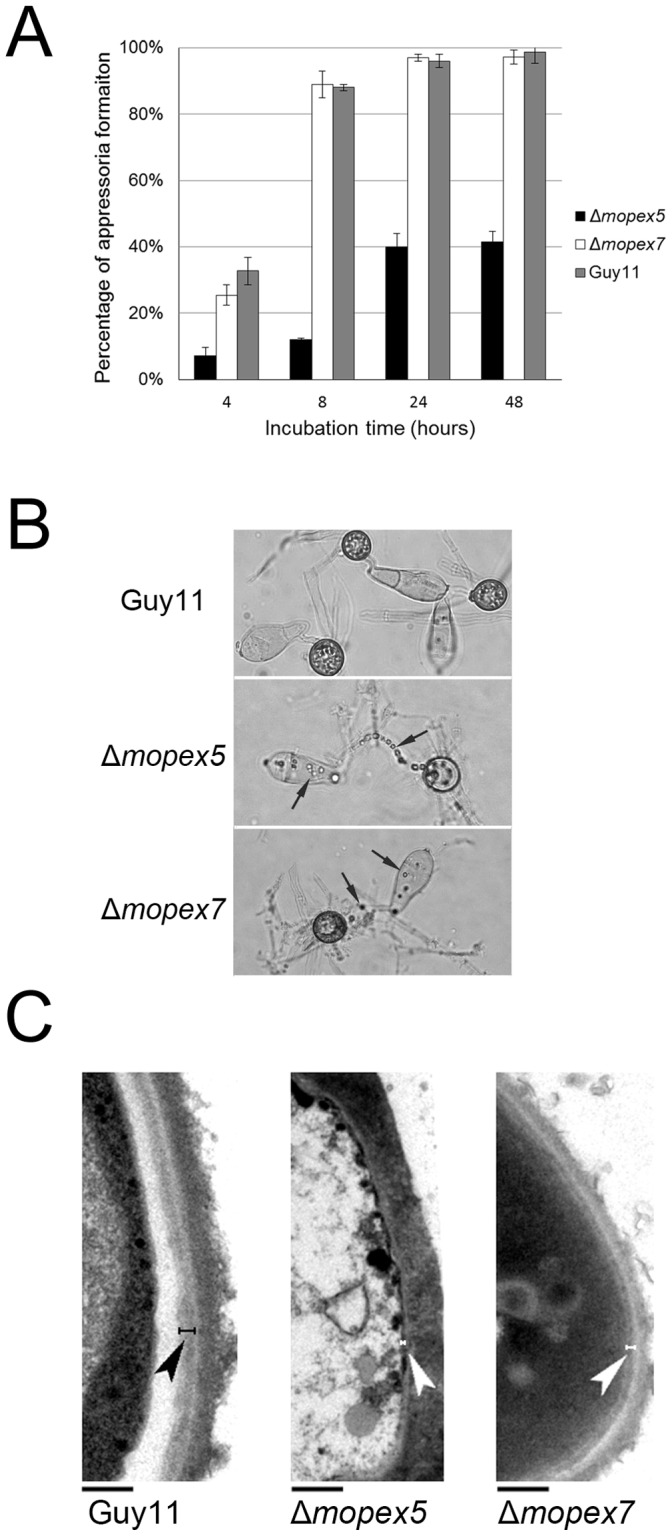
Appressorial morphology of the wild type, Δ*mopex5* and Δ*mopex7*. (A) Under the light microscope, the 24 h appressoria of Δ*mopex5* were less pigmented than the wild type and Δ*mopex7*, and numerous residual droplets (indicated by the arrows) presented in the conidia and germ tubes Δ*mopex5* and Δ*mopex7* the residues. (B) TEM analysis of 24 h appressoria. The melanin layers (indicated by the arrows) were detected in the wild type and Δ*mopex7* but absent in Δ*mopex5*. Bars = 0.5 µm.

Compared with the wild type and Δ*mopex7*, the ability of appressorial formation of Δ*mopex5* was reduced significantly. Induced for 24 h, only 22% germinated conidia of Δ*mopex5* formed appressoria, in contrast to the 97% of the wild type and Δ*mopex7* (Figure 6A). Under the light microscope, the appressoria of Δ*mopex5* could be found less pigmented than those of Guy11 (Figure 6B). For further confirmation, we performed the transmission electron microscopy (TEM). In 24 h appressoria of wild-type and Δ*mopex7*, a distinct melanin layer was detected between cell wall and plasma membrane as described previously [25]. But in Δ*mopex5*, this melanin layer was absent (Figure 6C). Together with the lighter color of the colony and liquid culture of Δ*mopex5*, these data indicated that *MoPEX5* was involved in melanin synthesis in mycelia and appressoria.

**Figure 7:**
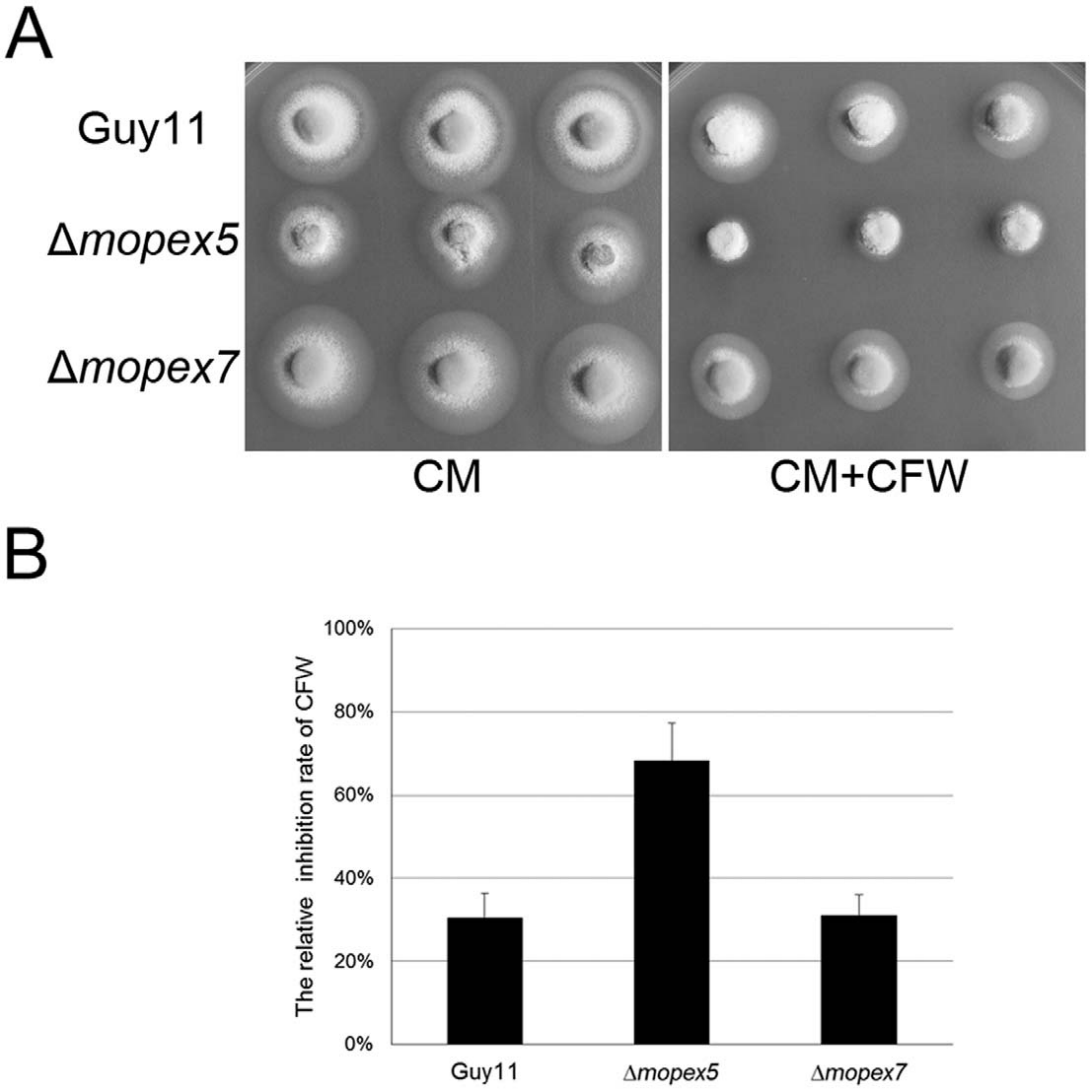
Cell wall integrity test of the wild type, Δ*mopex5* and Δ*mopex7*. The 5-mm mycelia discs of the strains were cultured on CM or CM supplied with 30 ug/ml Calcofluor white (CM+CFW) for 4 d, then the colonial diameters were measured and the relative inhibition rates of CFW to vegetative growth were calculated. (A) The colonies of the strain cultured for 4 d. (B) the relative inhibition rate of Δ*mopex*5 was higher significantly than that of the wild type and Δ*mopex7*. The relative inhibition rate (%) = [the colonial diameter (DIC) on CM – DIC on (CM+CFW)]/(DIC on CM –5).

Cell wall integrity is another key factor in addition to melanization to maintain appressorial function. To compare the cell wall integrity, the sensitivities of the strains to Calcofluor white (CFW), a cell wall-perturbing agent that binds to cellulose and chitin, were tested. Compared with the wild type, the sensitivity of Δ*mopex5* to CFW was increased (Figure 7A&B), indicating its cell wall was weakened. In addition, the tolerance of Δ*mopex5* to sodium dodecyl sulfate (SDS) was also reduced than Guy11, indicating a possible role of *MoPEX5* in integrity of cell membrane (Figure S2). Taking together, we concluded that *MoPEX5* was involved in the appressorial formation, appressorial melanization and integrity of cell wall and cell membrane; and the reductions of Δ*mopex5* in these aspects were the second reason for its infectional defects.

### MoPEX5 Participate in Lipid Mobilization and Appressorial Turgor Generation

**Figure 8:**
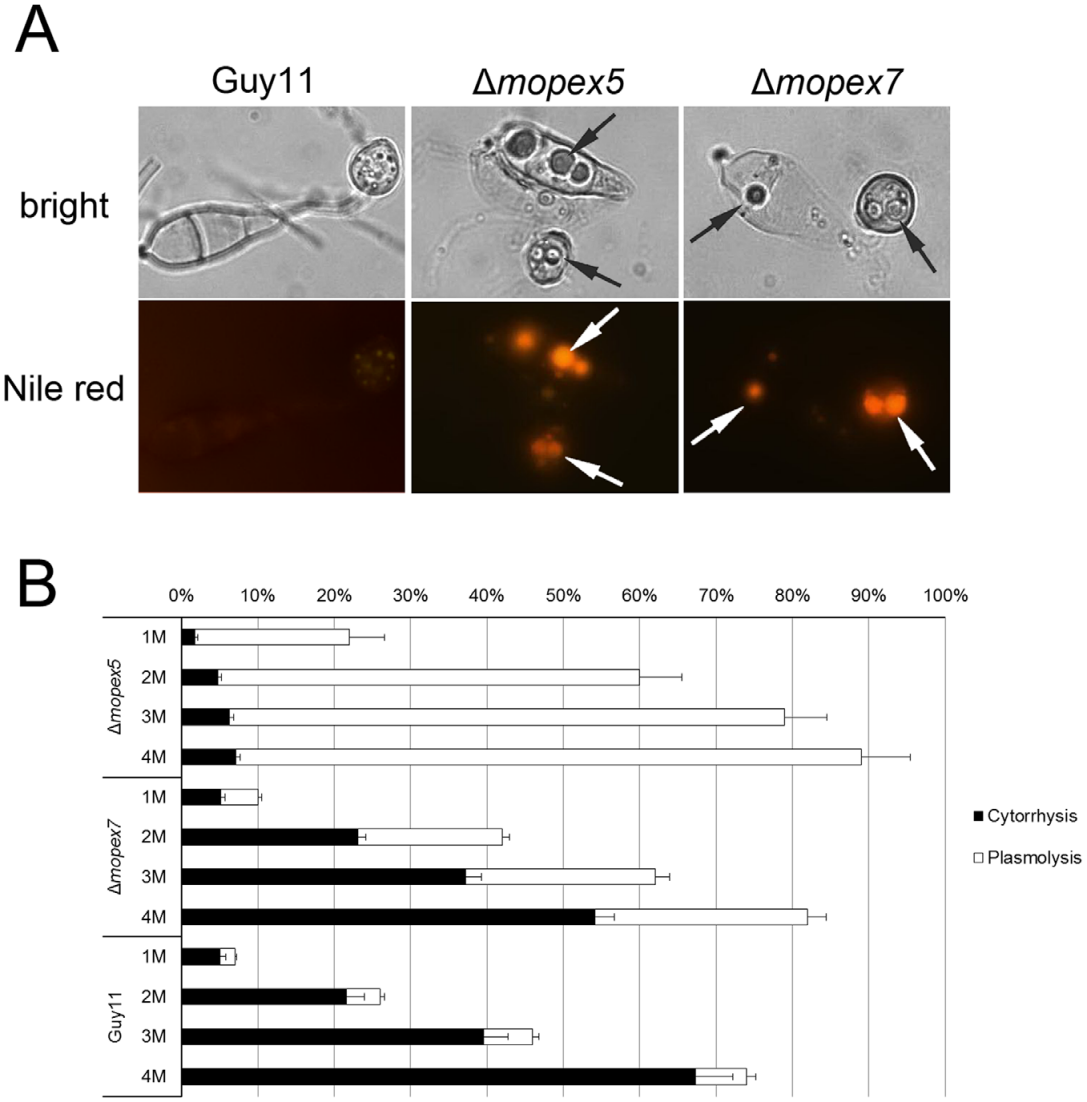
The lipids mobilization and turgor genesis of the wild type, Δ*mopex5* and Δ*mopex7*. (A) Numerous lipid residuals (indicated by the arrows) in the 24 h appressoria of Δ*mopex5* and Δ*mopex7* were visualized by Nile red staining. To improve the staining efficiency, 10 ppm tricyclazole was added to the conidial suspensions [65]. (B) cytorrhysis and plasmolysis test of 24 h appressoria. The appressoria were soaked in gradient concentrations of Glycerol. The percentages of cytorrhysis and plasmolysis occured were counted and statistically compared. The error bars on the top of the white columns represent standard deviations of plasmolysis.

In the wild type conidia induced for 24 h, the conidial contents were degraded completely and transferred into the appressoria, and then the conidia turned hollow and wizened. While in Δ*mopex5*, as well as Δ*mopex7*, numbers of droplets remained in appressoria, germ tubes and conidia kept undegraded even after 48 h (Figure 6B). These droplets were capable of staining by Nile red, indicating they were undegraded lipids (Figure 8A). In contrast, only very weak fluorescence was detected in the wild type which presented only in appressoria but not in conidia or germ tubes. Taking together with the result of lipid utilization, we concluded that the lipids degradation and their mobilization from conidia to appressoria were blocked in Δ*mopex5*, as well in Δ*mopex7* mutants.

Defective melanization, weakened cell wall and inadequate lipids conversion might influence the glycerol accumulation and turgor-genesis in appressoria. By using the cytorrhysis/plasmolysis test [28], we measured the appressorial turgor. Dipped in glycerol solutions, the appressoria of each strain exhibited both cytorrhysis and plasmolysis (Figure 8B). In the wild type, the plasmolysis occurred much less than cytorrhysis. However in Δ*mopex5*, the percentage of plasmolysis was improved significantly, exceeding that of cytorrhysis greatly. This result, corresponding with its missing melanin layer, indicated that the appressoria of Δ*mopex5* lost the glycerol impermeability and failed to maintain the turgor. The appressoria of Δ*mopex7* remained the ability to arrest glycerol where cytorrhysis occurred slightly more than plasmolysis. Nevertheless, the ratio of plasmolysis/cytorrhysis in Δ*mopex7* was higher than that of the wild type, indicating the cell wall integrity of its appressoria was also reduced, although no visible difference was found in its melanin layer and CFW resistance. At each glycerol concentration, the total percentages of cytorrhysis-occurred appressoria plus plasmolysis-occurred ones of each mutant were higher than those of the wild type, indicating the glycerol accumulation and appressorial turgor were reduced in both mutants. These reductions were possibly resulted from the blockage of lipids degradation and mobilization. The appressorial turgor was related directly to fungal penetration, thus its reduction was regarded as the third reason for the infectional defects of Δ*mopex5*, as well Δ*mopex7*.

### Glucose Supplement or Host Cuticle Removal Improved the Virulence of Δmopex5

**Figure 9:**
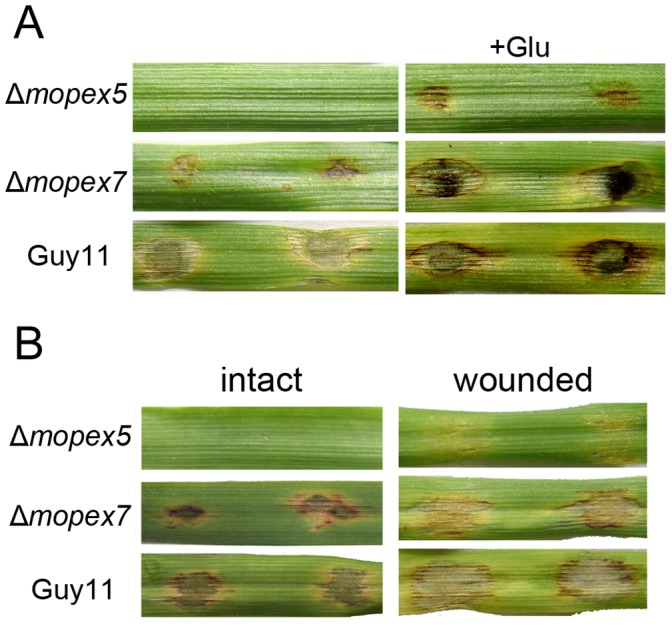
The virulence of Δ*mopex5* and Δ*mopex7* were offset partially by supplement with glucose or cuticle removal. (A) The intact leaves of barley ZJ-8 were droplet inoculated with conidial suspensions in 1×10^5^ conidia/ml supplemented with 2.5% glucose on intact leaves of Barley ZJ-8. The symptoms were recorded at 5 d post inoculation. (B) Conidial suspensions in 1×10^5^ conidia/ml were droplet inoculated on the intact and wounded leaves of Barley ZJ-8. The symptoms were recorded at 5 d post inoculation.

The lipids reserved in conidia is an important source of appressorial melanin, cell wall and glycerol, therefore the infectional defects of Δ*mopex5* were possibly resulted from defects in the lipids conversion. To confirm this deduction, we tested the effects of additional carbon source to virulence of Δ*mopex5* and Δ*mopex7*. Being supplemented with Glucose in conidial suspensions, both mutants offset their pathogenic defects partially. Δ*mopex5* generated visible lesions and Δ*mopex7* accelerated its symptom development (Figure 9A). Taking together, we concluded that the incomplete lipid degradation in Δ*mopex5* caused nutrition insufficiency, which led to the defective melanization, weakened cell wall and reduced glycerol accumulation, and finally resulted in the defects in cuticle penetration.

To learn whether the genes functioned also at post-penetration stage, droplet inoculation was conducted on wounded barley leaves whose cuticles were removed (Figure 9B). In contrast to the intact leaves where Δ*mopex5* didn’t cause any lesions, the wounded leaves inoculated with Δ*mopex5* generate slight symptoms. Also, Δ*mopex7* caused more advanced lesions on wounded leaves than on intact ones. This result indicated that the virulence defects of Δ*mopex5* and Δ*mopex7* were partially restored by removing the cuticles, corresponding with the involvement of *MoPEX5* and *MoPEX7* in cuticle penetration. However, despite on wounded leaves, the lesions derived from Δ*mopex5* or Δ*mopex7* were still less developed than those from the wild type. Combined with the histological observation of infection, the result indicated that *MoPEX5*, as well as *MoPEX7*, played roles in fungal development within plant tissue in addition to the cuticle penetration.

### Δmopex5 and Δmopex7 Mutants were both Hypersensitive to H_2_O_2_

**Figure 10:**
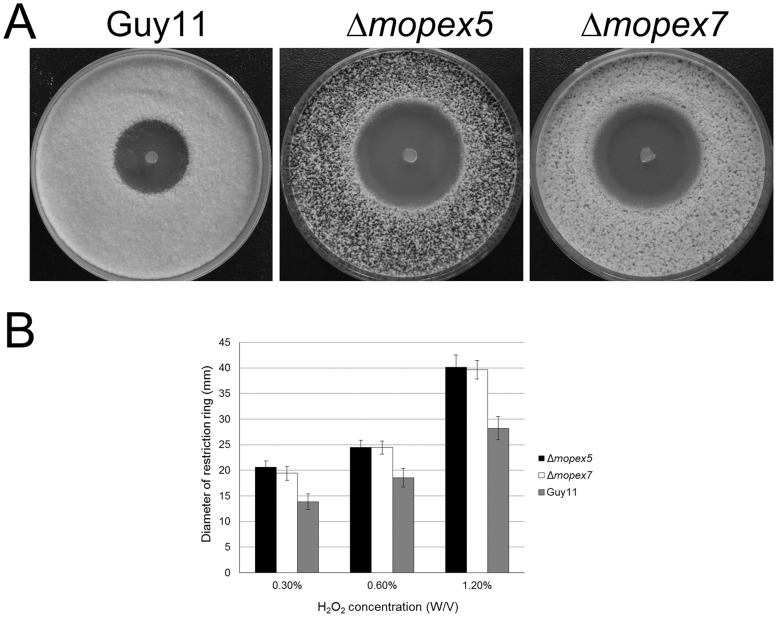
Suppression of H_2_O_2_ to the conidial germination of Δ*mopex5*, Δ*mopex7* and the wild type. (A) the medium discs containing 0.5 M H_2_O_2_ formed larger restraining rings on the germinated conidia of Δ*mopex5* and Δ*mopex7* than those of the wild type; (B) The diameters of the restraining rings on the strains were measured and statistical compared.

Since neither the glucose supplement nor the cuticle removal restored the full virulence of the mutants, the defects in lipids degradation and cuticle penetration were unable to fully explain the infection failure. In view of that ROS in host cells are a main barrier for fungal invasion while ROS degradation is another main metabolism in peroxisomes, we tested the abilities of the strains to degrade H_2_O_2_, a main form of ROS. On CM plates, the conidial germinations were restrained by H_2_O_2_ and formed restraining rings. The rings formed by Δ*mopex5* and Δ*mopex7* showed no difference in between, but were larger significantly than that of the wild type (Figure 10A&B). Similarly, the H_2_O_2_ tolerance of Δ*mopex5* and Δ*mopex7* during vegetative growth were also reduced significantly in comparison with the wide type (Figure S3). These data indicated that the abilities of H_2_O_2_ degradation of Δ*mopex5* and Δ*mopex7* were reduced. The pathogens must overcome ROS in host cells to invade smoothly. The reduced ability of ROS degradation was thus a possible reason for why the mutants slowed down the growth of their infectious hyphae in host tissue and developed lower symptom despite on wounded leaves or with supplemented Glucose. Taking the phenotypic data together, we concluded that the infection failure of the mutants was resulted not only from the penetration defects caused by the insufficient lipid degradation, but also the developmental defects in host tissue caused by the decrease resistance to ROS.

### Gene Reintroduction Restored the Virulence of the Corresponding Mutants

**Figure 11:**
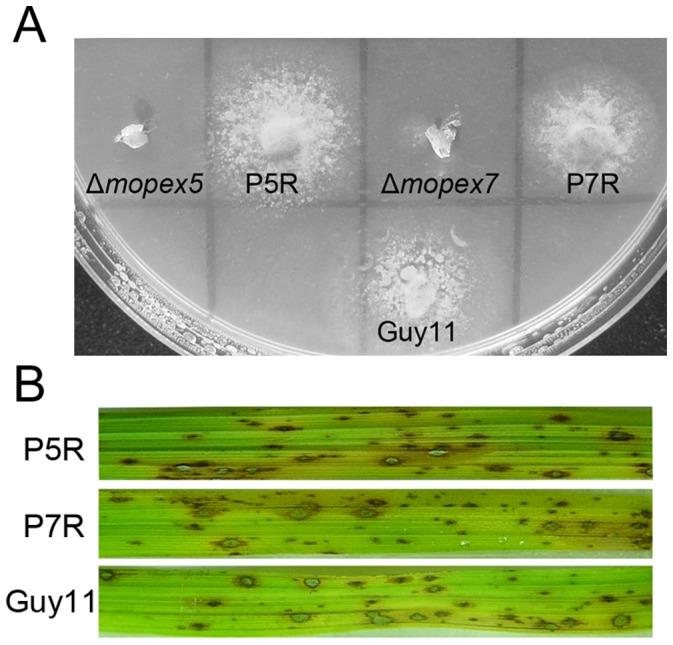
Complementation of Δ*mopex5* and Δ*mopex7* mutants by reintroduction of *MoPEX5* and *MoPEX7* genes. (A) The Δ*mopex5* and Δ*mopex7* mutants, the wild strain Guy11 and complemented strains of Δ*mopex5* (P5R) and Δ*mopex7* (P7R) were cultured on minimal medium with olive oil as sole carbon source. (B) Leaves from rice plant of cultivar CO39 inoculated with conidial suspensions of the wild type, and complemented strains P5R and P7R.

To ensure that the pathogenicity-related changes in Δ*mopex5* and Δ*mopex7* were associated truly with the genes disruption events, the *MoPEX5* and *MoPEX7* genes were reintroduced into their respective mutants. Transformants carried a single-copy integration were selected and ensured by Southern blot analysis. The complementary transformants of *MoPEX5* (P5R) and *MoPEX7* (P7R) were all able to grow on the media with lipid as sole carbon source and exhibited full virulence (Figure 11A&B). We concluded that the deletion of *MoPEX5* and *MoPEX7* were responsible for the virulence defects and the related phenotypes in *M. oryzae*.

### MoPEX5 and MoPEX7 were Highly Expressed in Conidia and during Conidial Germination

**Figure 12:**
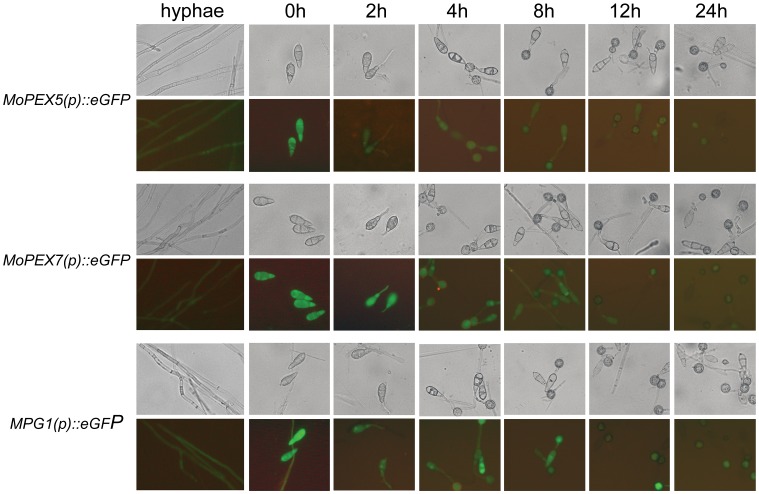
Expression of *MoPEX5* and *MoPEX7* during conidial germination and appressorium development. Promoter regions of *MoPEX5*, *MoPEX7* and were respectively fused to the GFP green fluorescent protein-encoding allele and introduced into *M. oryzae* strain Guy 11. Transformants carrying single-copy integrations were identified. Fluorescence of vegetative hyphae and during the appressorium development of germinating conidia on a hydrophobic surface of the transformants were analyzed and compared by epifluorescence microscopy. Control experiment in which a transformant expressing GFP under control of *MPG1* [34] gene was allowed to undergo appressorium formation for 24 h.

In order to investigate the temporal and spatial patterns of *MoPEX5* and *MoPEX7* expression during infection-related development, 1.5 kb promoter fragments upstream of *MoPEX5* and *MoPEX7* protein-coding sequences were fused respectively to the green fluorescent protein-encoding gene *GFP*. The fusions were introduced into the wild type strain Guy 11, and the transformants with a single integration of the plasmid were selected for fluorescent observation. As a control, a 1.6 kb promoter fragment of *MPG1*, a strongly expressed gene in rice blast fungus [34], was also fused to *GFP* and introduced into Guy11. Both hyphae and conidia, harvested from plate cultures of *MoPEX5(p):GFP* and *MoPEX7(p):GFP* transformants, exhibited GFP fluorescence, but the fluorescence from the conidia were in much higher levels (Figure 12). When the conidia of the transformants were incubated on inductive membrane to allow germination and appressoria formation, the expression of both *MoPEX5(p):GFP* and *MoPEX7(p):GFP* declined gradually. At the initiation phase of this process, namely, during conidia germination, germ tubes elongation and initial emergence of appressoria, the fluorescence of *MoPEX5(p):GFP* and *MoPEX7(p):GFP* were still in fairish levels. But subsequently, along with the swelling, melanization and maturation, the fluorescence was weakened incessantly and almost absent in 24 h mature appressoria, in contrast with those of *MPG1(p):GFP* which kept continuous high expression during this process. The results indicated that *MoPEX5* and *MoPEX7* had similar expression patterns, namely, a high expression in conidia and during germination and subsequent decline, which were also confirmed by quantifying the transcripts at each time points using quantitative-PCR (Figure S4). The expression patterns, corresponding with the number variation of peroxisome we previously described [32], hinted a high requirement of peroxisomal metabolism during conidial germination.

## Discussion

The characterization of peroxisomal biogenesis related genes (*PEXs*) and peroxisomal metabolism related genes demonstrated that the peroxisomes are required in fungal pathogenicity [23], [25], [45]. However, the peroxisomal biogenesis machinery is so complicated that different genes in same organism or homologous genes in different species may play different roles [27], [46], [47]. Here, we focused on the shared and distinct contributions of *MoPEX5*, the predicted PTS1 receptor encoding gene, to the PTS2 receptor encoding gene, *MoPEX7*, in fungal development and pathogenicity of *M. oryzae*. The result indicated that *MoPEX5* participated specifically in PTS1 pathway. Both *MoPEX5* and *MoPEX7* are required in multiple aspects in fungal development and pathogenicity, and comparatively, *MoPEX5* played more roles.

### MoPEX5 and MoPEX7 were Involved Respectively in PTS1 and PTS2 Pathways

Although the import machinery of peroxisomal matrix proteins are generally conserved, differences present among organisms [14], [16], [19], [46], [48]. An intriguing example is the cross-talking between PTS1 and PTS2 pathways. In yeasts, *PEX5* and *PEX7* play roles independently, i.e., *PEX5* serves only PTS1 and *PEX7* serves only PTS2 [9], [10], [49], [50], [51], and in these cases, an accessary protein, Pex20p (or Pex18p/Pex21p) participates in PTS2 pathway as a co-receptor to Pex7p [46], [52]. However in *Arabidopsis*, Pex5p contributes to import of both PTS1 and PTS2 containing proteins [16], [17]. And in mammals and rice, *PEX5* generates two splicing isoforms, Pex5pS and Pex5pL [15], [18], [19], [20]. The former binds PTS1, whereas the latter mediates the import of both PTS1 and PTS2 via interaction with Pex7p [53], [54]. Mammals and rice lack Pex20p but have a Pex20p-like motif in their Pex5pL, which might mediate the interaction between Pex5pL and Pex7p [14], [55], [56]. In ascomycetes, the presence of the Pex20p and the absence of Pex20-like motif in their Pex5p predicted the independence of their *PEX5* to the PTS2 pathway [21], [47]. But this prediction still needs more experimental evidence and whether PTS1 and PTS2 are coupled in ascomycetes is unclear yet. Here, the import of PTS1 and PTS2 in Δ*mopex5* and Δ*mopex7* demonstrated that *MoPEX5* and *MoPEX7* worked independently, resembling the scenario in yeasts. Correspondingly, neither alternative splicing nor Pex20-like motif was detected in *MoPEX5* by cDNA sequencing and motif searching. Meanwhile, a conserved Pex20p orthologue (MGG00638) was found in the *M. oryzae* genome. These data indicated that *PEX5* and *PEX7* function independently in ascomycetes.

### The Involvement of Peroxisome in Fungal Infection Related to Lipids Metabolism and ROS Degradation

The Δ*mopex5* mutant, together with Δ*mopex7* and Δ*mopex6*, demonstrated the involvement of the peroxisomes in multiple pathogenicity-related events, such as conidiation, appressorial morphogenesis and hyphal development in host tissue [23], [25], [30]. Peroxisomes participate widely in various metabolisms, of which, the lipids degradation and ROS removal are conserved among different organisms. As known, the conidia of *M. oryzae* have no exogenous nutrition to use and have to degrade their own storage reserves to supply all the material and energy for infection morphogenesis, while lipid is one of the main reserves and its importance to fungal infection has been demonstrated [29], [57], [58]. In eukaryotes, lipids are metabolized by two peroxisomal processes, β-oxidation and glyoxylate cycle, to produce acetyl-CoA [3]. Acetyl-CoA is an important molecule which can be used to generate ATP in TCA cycle or convert to glucose via gluconeogenesis. The resultant Glucose can be further converted to glycerol, as well chitin and β-1, 3-glucan, the components of fungal cell wall. Additionally, Acetyl-CoA is also the starter and extender units in fungal melanin synthesis. Thus, lipids are an important source for melanin, glycerol, cell wall and energy (ATP), the key factors for fungal infection. Δ*mopex5* and Δ*mopex6* exhibited defects in all these factors, and as well Δ*mopex7* in glycerol and cell wall [23], [25], [30]. And meanwhile, Δ*mopex5*, Δ*mopex6* and Δ*mopex7* were defective in lipids utilization and in lipids mobilization from conidia to appressoria. Moreover, the supplemented glucose could partially offset the pathogenicity of the mutants. These facts indicated that the contributions of the peroxisome to fungal pathogenicity were related, to a great extent, to lipids degradation. Correspondingly, the *MoPEX5* and *MoPEX7* genes were highly expressed during the conidial germination and initial emergence of appressoria, which are just the main period of lipid degradation [25]. This deduction can also be confirmed by the mutations of the key genes in β-oxidation and glyoxylate cycle which caused some shared defects as the *pex* mutants to the fungus [23], [45], [58].

On the other hand, the host cells produce abundant ROS to suppress pathogen infection. The pathogens have to remove these excess ROS for survival. Δ*mopex5* and Δ*mopex7* showed slowed hyphal growth in host cells and meanwhile the decreased resistance to H_2_O_2_, implying that the reduction of ROS degradation was responsible, at least partially, for the defects in post-penetration phases. To analyze the roles of the enzymes in ROS degradation, such as catalase and peroxidase, in fungal infection, is thus interesting and will improve the understanding of this topic. In addition to lipids and ROS metabolism, the other peroxisomal metabolic reactions also have the possibility to affect the fungal pathogenicity, for example, a hypothetic multicopper oxidase containing PTS1 showed closely similarity to Abr1p, a protein involved in melanin biosynthesis in *Aspergillus fumigatus* [59].

### PTS1 and PTS2 Pathways Contribute Differently to Fungal Pathogenicity

Since the symptom is an accumulated result of pathogenicity-related factors, the levels of virulence decrease are determined by the numbers and the levels of the factors affected by gene deletion. *MoPEX5* was involved in more pathogenicity-related factors than *MoPEX7*, thus Δ*mopex5* lost entirely pathogenicity while Δ*mopex7* did not. To find the clues to the predominance of *MoPEX5* in pathogenicity, we investigated the PTSs presented in the key enzymes of lipids degradation. In the 33 hypothetic enzymes collected from *M. oryzae* genome, 16 contained typical PTSs, and of which, 13 were PTS1 and three were PTS2 (Table S1). The presences of both PTS1 and PTS2 in the enzymes corresponded to the requirement of both *MoPEX5* and *MoPEX7* in lipids metabolism. Meanwhile, PTS1 presented predominantly in the enzymes, implying that *MoPEX5* probably contributed more to lipids metabolism than *MoPEX7*, although their difference was not detected in lipid utilizing experiment. This is maybe the reason for why more related phenotypes, namely, melanization, cell wall biogenesis and glycerol accumulation, were affected in Δ*mopex5*, while in Δ*mopex7*, only the glycerol accumulation. This phenomenon inferred that the fungus likely ensured its melanization preferentially to glycerol accumulation when facing to nutrient starvation. Additionally, some other PTS containing proteins in *M. oryzae* could make better understanding for the phenotypes of Δ*mopex5* and Δ*mopex7.* For example, a PTS1- (SKL) containing protein, MGG05138, was a homologue of *SPS19* which was required in the vegetative growth and sporulation of *S. cerevisiae* and *N. crassa* [60], [61]. A multicopper oxidase, MGG07220, which presented a PTS1 (AKL), showed similarity to Abr1p, an enzyme involved in melanin biosynthesis [59]. This finding hinted a possibility that the melanization defects in Δ*mopex5* were derived also from melanin synthesis itself in addition to the nutrient starvation. That is maybe the reason for why Δ*mopex5* still lacked melanin in rich media (CM) and why Δ*mopex7* was unaffected in melanization despite its lipids metabolism was disordered.

But remarkably, more than a half of these enzymes had no PTS1 or PTS2. One possibility of this fact is that the metabolisms known as occurred dominantly in peroxisomes could also performed in cytoplasm or other organelles; another possibility is that these enzymes has special PTS which could be recognized by *PEX5*, *PEX7* or even novel receptors in import pathways unknown so far. The possibility of the existence of new import pathways was demonstrated by *PEX20* in *P. anserine* which mediated the import of peroxisomal matrix proteins independently for some specific developmental processes [46].

To reveal the combined effects of *MoPEX5* and *MoPEX7*, we tried double deletion but failed to obtain double mutants after screening more than 300 transformants. Thus, the double deletants of the two genes is maybe lethal in *M. oryzae*. Nevertheless, *MgPEX6*, which was involved in both PTS1 and PTS2 pathways, gave some clues to the synergic effects of *MoPEX5* and *MoPEX7* [23], [25]. The Δ*mgpex6* mutant lost the pathogenicity completely and exhibited slower vegetative growth, reduced appressorial melanization and decreased conidiation, resembling closely to Δ*mopex5*, and thus indicating that the defects of Δ*mgpex6* were related mostly to its disorder in PTS1 pathway. But the damages enhanced by the disorder of PTS2 pathway could also be found in Δ*mgpex6* mutant. For example, the additional glucose offset the virulence of Δ*mopex5*, despite in a low level, but failed to do that in Δ*mgpex6*. Thus, the phenotypes of Δ*mgpex6* reflected in some extent the defects derived from disorder of both PTS pathways. Despite that, the phenotypes of Δ*mgpex6* could not be simply regarded as a summation of Δ*mopex5* and Δ*mopex7*, because no evidence so far indicated *PEX6* could control all the functions of *PEX5* and *PEX7*. Therefore, it would be very interesting to further clarify the mechanism and the roles of import of peroxisomal matrix proteins, including new ones such as that mediated by *PEX20* [46], as well as the import of peroxisomal membrane proteins which was mediated by *PEX19* [62].

In addition, some slight differences could be found between the *MoPEX7* mutants derived from Guy11 and from KJ201 [30]. For example, the Δ*mopex7* from KJ201 exhibited dramatic decrease in conidiation while that from Guy11 did not. And some other quantitative differences were also found in between. Such differences derived from strain backgrounds could also be found when we compared the parallel studies of Δ*pth2*, Δ*mgpex6* and Δ*hex1* [23], [25], [63], [64]. This reminds us that investigating a gene in more strains would possibly make more comprehensive knowledge. Nevertheless, the slight differences did not affect the final conclusion. Taken together, our studies indicated that *MoPEX5* and *MoPEX7* mediate PTS1 and PTS2 peroxisomal pathways respectively and independently. Both PTS1 and PTS2 pathways played multiple roles in fungal development and pathogenicity, with the PTS1 pathway playing a more predominant role than the PTS2 pathway.

## Supporting Information

Figure S1 **Vegetative growth of Δ** ***mopex5*** **, Δ** ***mopex7*** **and wide type on medium with sodium acetate as sole carbon source.**
(TIF)

Figure S2 **Tolerance to SDS of the Δ** ***mopex5*** **, Δ** ***mopex7*** **and the wild type.** The 5 mm mycelia discs were cultured on CM, CM supplied with 0.01% SDS or 0.02% SDS for 8 d, then the colonial diameters were measured and the relative inhibition rates were calculated. (A) The colonies of the strain cultured for 8 d. (B) the relative inhibition rate of Δ*mopex*5 was higher significantly than those of the wild type and Δ*mopex7*. The relative inhibition rate (%) = [the colonial diameter (DIC) on CM – DIC on (CM+SDS)]/(DIC on CM –5).
(TIF)

Figure S3 **Tolerance to H_2_O_2_ of** Δ***mopex5*** **,** Δ***mopex7*** **and the wild type during vegetative growth.** The 5 mm mycelia discs were cultured on CM, CM supplied with 2.5 mM or 5 mM H_2_O_2_ for 10 d, then the colonial diameters were measured and the relative inhibition rates were calculated. (A) The colonies of the strain cultured for 10 d. (B) the relative inhibition rates of Δ*mopex*5 and Δ*mopex7* were higher significantly that of the wild type. The relative inhibition rate (%) = [the colonial diameter (DIC) on CM – DIC on (CM+H_2_O_2_)]/(DIC on CM –5).
(TIF)

Figure S4 **Relative expression of** ***MoPEX5*** **and** ***MoPEX7*** **during appressoria development.** The conidia were allowed to form appressoria on a hydrophobic surface and sampled at different culture time points. The relative expressions of *MoPEX5* and *MoPEX7* in each sample were analyzed by using quantitative-PCR.
(TIF)

Table S1 **Predicted PTSs in enzymes involved in lipids degradation of** ***M. oryzae*** **.** The open reading frames (ORF) encoding key enzymes involved in lipids degradation were first identified by searching the enzyme names against the *M. oryzae* genome database (http://www.broadinstitute.org/annotation/genome/magnaporthe_comparative/MultiHome.html), and then used as core sequences to re-search against the database using blastP procedure to get more potential candidates. The candidates were then checked in the NCBI database using blastP procedure to pick out the ORFs predicted to encode the enzymes. The PTS1 motifs were predicted by the PTS1 predictor (http://mendel.imp.ac.at/mendeljsp/sat/pts1/PTS1predictor.jsp) with the parameter FUNGI-specific function [66], [67]. The PTS2 motifs were predicted by searching the consensus (R/K)-(L/V/I)-X5-(H/Q)-(L/A) [13].
(DOC)
