# Supplementary figures and images for: PTS1 Peroxisomal Import Pathway Plays Shared and Distinct Roles to PTS2 Pathway in Development and Pathogenicity of Magnaporthe oryzae
Source: PLoS One. 2013 Feb 6;8(2):e55554. doi: 10.1371/journal.pone.0055554 (PMC3566003; doi:10.1371/journal.pone.0055554)

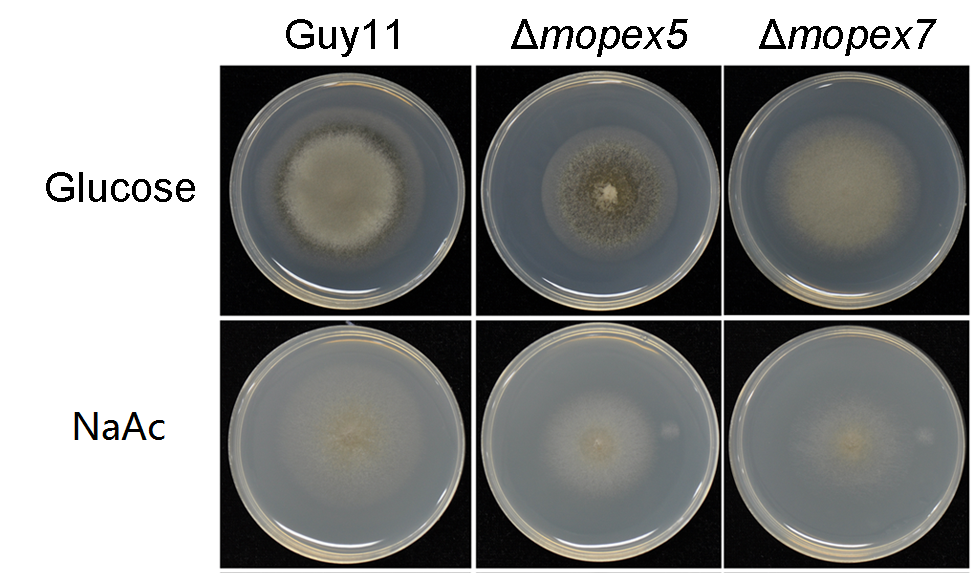

Supplement: Figure S1 — Vegetative growth of Δ mopex5 , Δ mopex7 and wide type on medium with sodium acetate as sole carbon source. (TIF) [file pone.0055554.s001.tif]

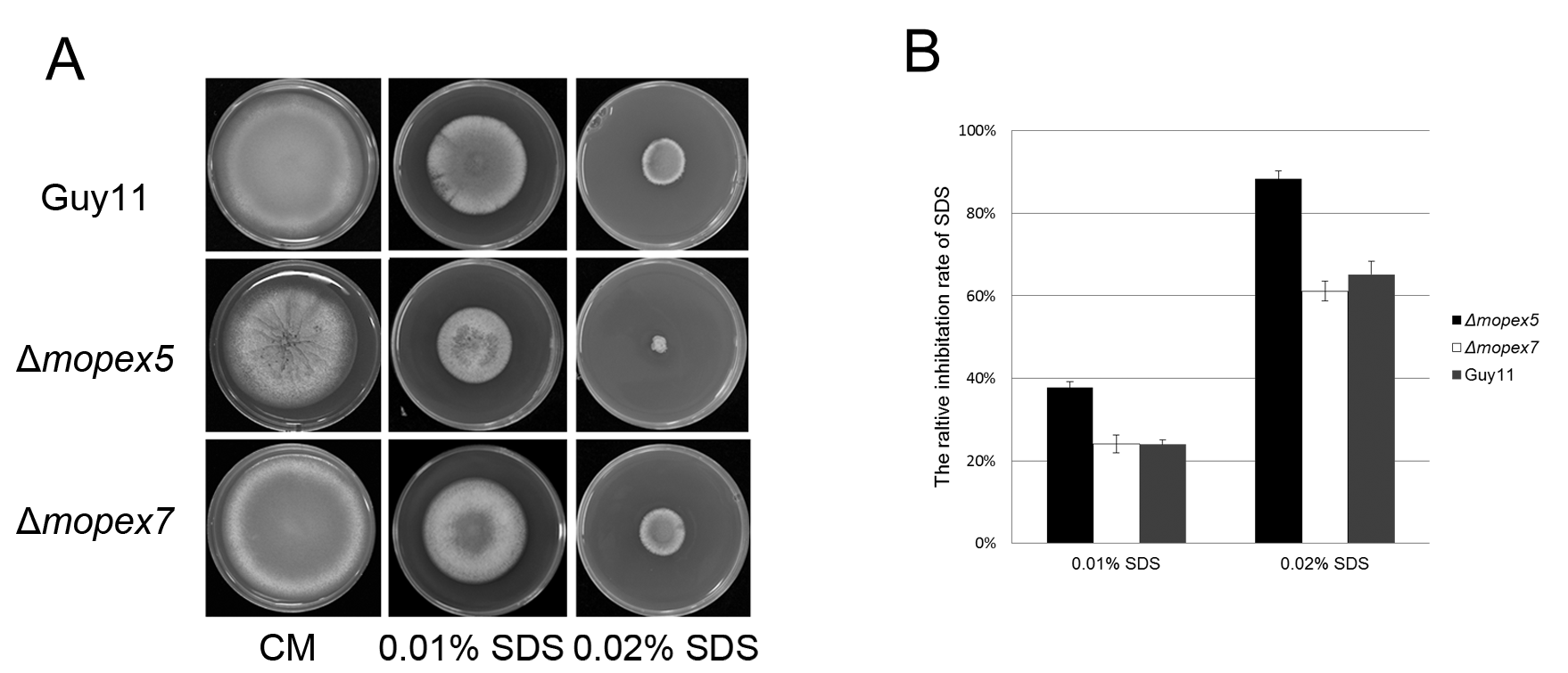

Supplement: Figure S2 — Tolerance to SDS of the Δ mopex5 , Δ mopex7 and the wild type. The 5 mm mycelia discs were cultured on CM, CM supplied with 0.01% SDS or 0.02% SDS for 8 d, then the colonial diameters were measured and the relative inhibition rates were calculated. (A) The colonies of the strain cultured for 8 d. (B) the relative inhibition rate of Δmopex5 was higher significantly than those of the wild type and Δmopex7. The relative inhibition rate (%) = [the colonial diameter (DIC) on CM – DIC on (CM+SDS)]/(DIC on CM –5). (TIF) [file pone.0055554.s002.tif]

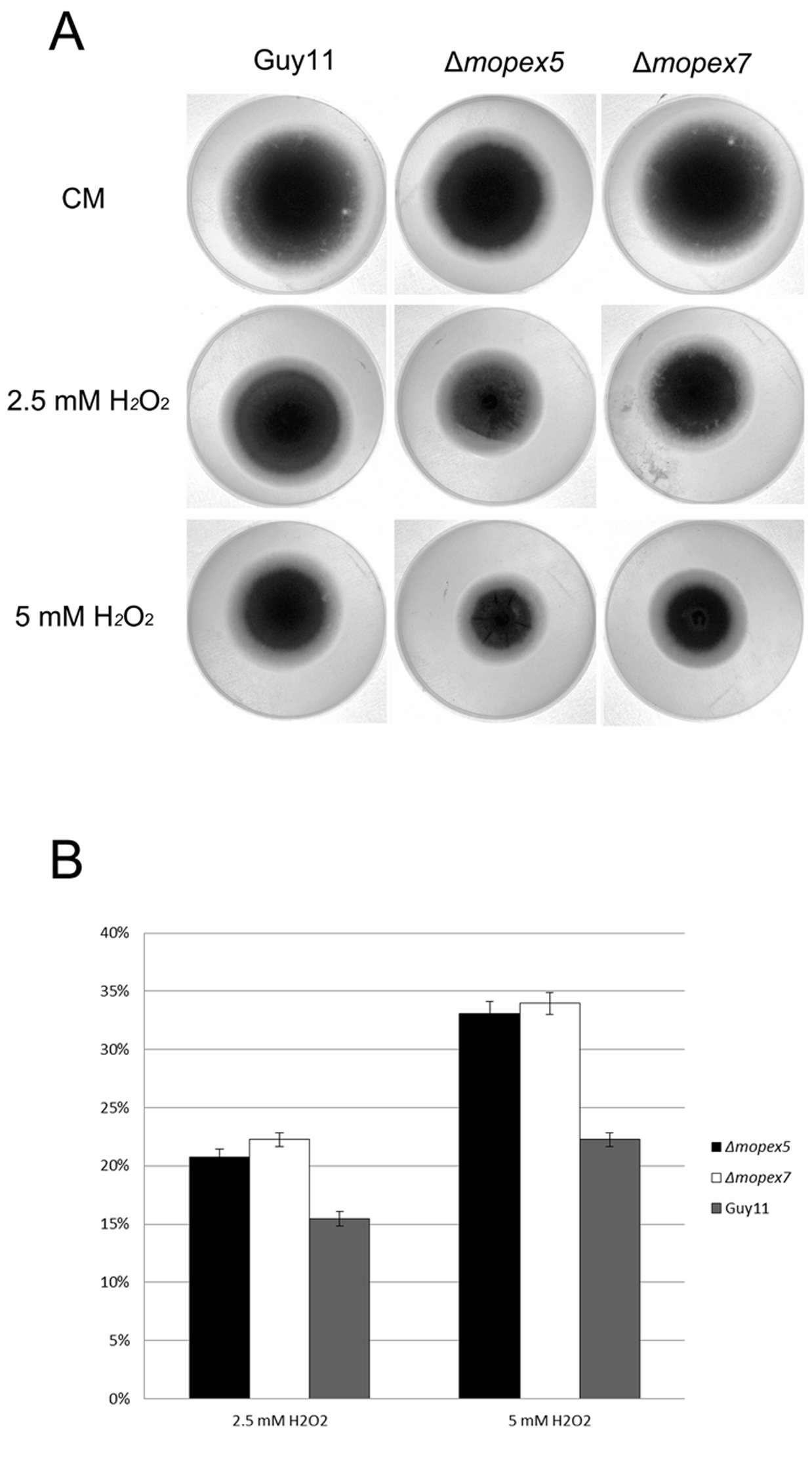

Supplement: Figure S3 — Tolerance to H2O2 of Δmopex5 , Δmopex7 and the wild type during vegetative growth. The 5 mm mycelia discs were cultured on CM, CM supplied with 2.5 mM or 5 mM H2O2 for 10 d, then the colonial diameters were measured and the relative inhibition rates were calculated. (A) The colonies of the strain cultured for 10 d. (B) the relative inhibition rates of Δmopex5 and Δmopex7 were higher significantly that of the wild type. The relative inhibition rate (%) = [the colonial diameter (DIC) on CM – DIC on (CM+H2O2)]/(DIC on CM –5). (TIF) [file pone.0055554.s003.tif]

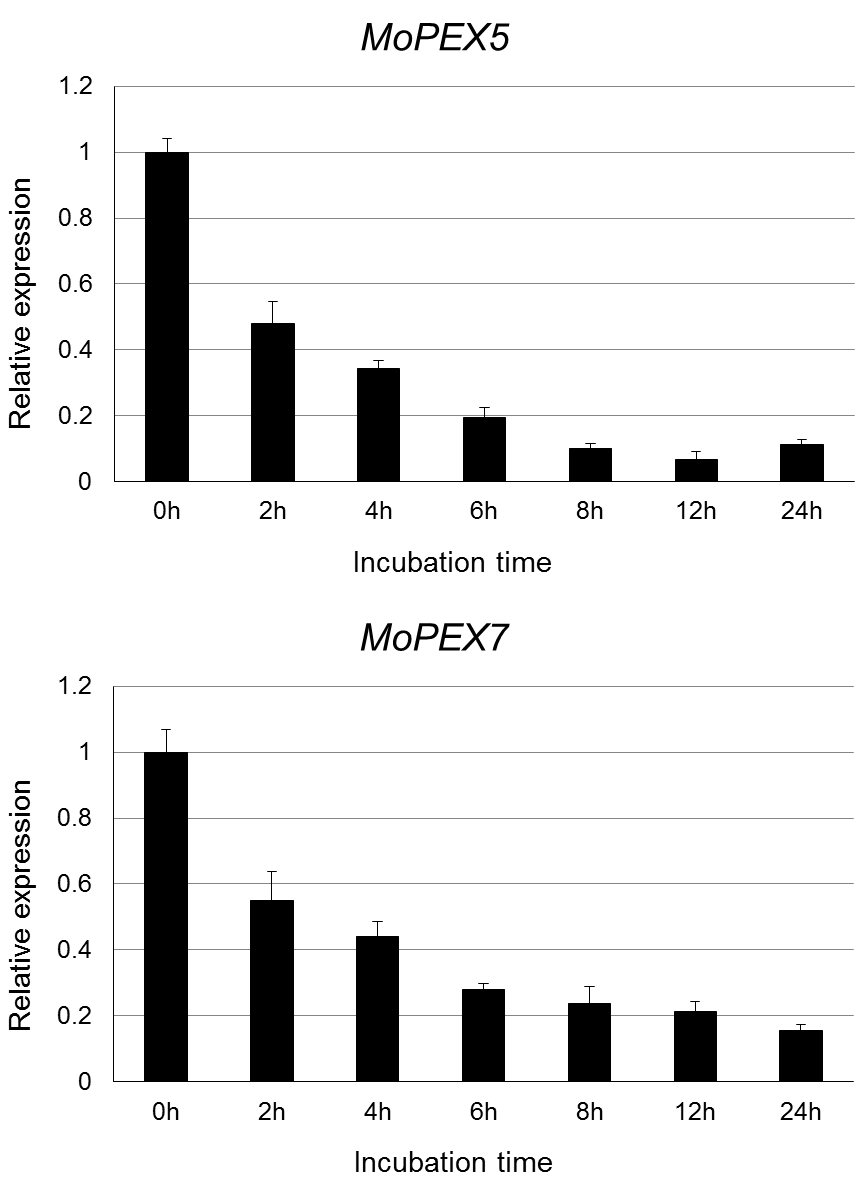

Supplement: Figure S4 — Relative expression of MoPEX5 and MoPEX7 during appressoria development. The conidia were allowed to form appressoria on a hydrophobic surface and sampled at different culture time points. The relative expressions of MoPEX5 and MoPEX7 in each sample were analyzed by using quantitative-PCR. (TIF) [file pone.0055554.s004.tif]
