# Supplementary material for: PTS1 Peroxisomal Import Pathway Plays Shared and Distinct Roles to PTS2 Pathway in Development and Pathogenicity of Magnaporthe oryzae
Source: PLoS One. 2013 Feb 6;8(2):e55554. doi: 10.1371/journal.pone.0055554 (PMC3566003; doi:10.1371/journal.pone.0055554)
Supplement: Table S1 — Predicted PTSs in enzymes involved in lipids degradation of M. oryzae . The open reading frames (ORF) encoding key enzymes involved in lipids degradation were first identified by searching the enzyme names against the M. oryzae genome database (http://www.broadinstitute.org/annotation/genome/magnaporthe_comparative/MultiHome.html), and then used as core sequences to re-search against the database using blastP procedure to get more potential candidates. The candidates were then checked in the NCBI database using blastP procedure to pick out the ORFs predicted to encode the enzymes. The PTS1 motifs were predicted by the PTS1 predictor (http://mendel.imp.ac.at/mendeljsp/sat/pts1/PTS1predictor.jsp) with the parameter FUNGI-specific function [66], [67]. The PTS2 motifs were predicted by searching the consensus (R/K)-(L/V/I)-X5-(H/Q)-(L/A) [13]. (DOC) [file pone.0055554.s005.doc]

**Supplement Table 1.** Predicted PTSs in enzymes involved in lipids degradation of *Magnaporthe oryzae*

| Predicted genes involved in fatty acid transfer | | | | |
| --- | --- | --- | --- | --- |
| No. | Name | Characters/functions | PTS | PTS related sequence |
|  | MGG06177 | Acyl-CoA-binding protein, intracellular carrier of acyl-CoA esters |  |  |
|  | MGG05025 | Long-chain fatty acids transporter protein 3, transport fatty acids across the membrane | I | 620 DWNALGVGKAKL 631 |
|  | MGG08257 | Long-chain fatty acid transporter, transport fatty acids across the membrane | I | 624 DWDGLKKGLVKL 635 |
|  | MGG01721 (*CRAT1, PTH2*) | Carnitine acetyl-transferase, transmembrane of acyl-CoA | I | 603 LMPTIEAPKSKL 614 |
|  | MGG06981 (*CRAT2*) | Carnitine acetyl-transferase, transmembrane of acyl-CoA |  |  |
| Predicted genes involved in fatty acid β-oxidation | | | | |
| No. | Name | Characters/functions | PTS | PTS related sequence |
|  | MGG04956 | Long-chain fatty acyl-CoA ligase, activation of long-chain fatty acids |  |  |
|  | MGG01551 | Long-chain fatty acyl-CoA ligase, activation of long-chain fatty acids |  |  |
|  | MGG07197 | Long-chain fatty acyl-CoA ligase, activation of long-chain fatty acids | I | 696 EINSQPVAKAKL 707 |
|  | MGG08288 | Short-chain-fatty-acid-CoA ligase, activation of short-chain fatty acids | I | 568 NKLIEDKVKAKL 579 |
|  | MGG02862 | Short-chain-fatty-acid-CoA ligase, activation of short-chain fatty acids |  |  |
|  | MGG09647 | Acyl-CoA synthetase, activation of fatty acids |  |  |
|  | MGG06199 | Peroxisomal-CoA synthetase, activation of fatty acids | I | 522 ETMQKQDNKAKL 533 |
|  | MGG7705 | Acyl-CoA ligase, activation of fatty acids |  |  |
|  | MGG08661 | Acyl-CoA dehydrogenase, catalyzes first step in fatty acid β-oxidation | I | 441 EPGTGITRKSKL 452 |
|  | MGG16316 | Acyl-CoA dehydrogenase, catalyzes first step in fatty acid β-oxidation | I | 452 GAGGDREERSKL 463 |
|  | MGG03418 | Acyl-CoA dehydrogenase, catalyzes first step in fatty acid β-oxidation |  |  |
|  | MGG05949 | Acyl-CoA dehydrogenase, catalyzes first step in fatty acid β-oxidation |  |  |
|  | MGG08690 | Acyl-CoA dehydrogenase, catalyzes first step in fatty acid β-oxidation, short-orbranched-chain specific |  |  |
|  | MGG15041 | Acyl-CoA dehydrogenase, catalyzes first step in fatty acid β-oxidation, medium-chain specific | I | 372 EHKVAKEREAKL 383 |
|  | MGG06148 (*MFP1*) | Multifunctional β-oxidation protein, catalyzes second and third steps in fatty acid β-oxidation | II | 78 NGDKIIDTAIQAFGR 92 |
|  | MGG12868 | Enoyl-CoA hydratase, catalyzes second step in fatty acid β-oxidation |  |  |
|  | MGG11223 | Enoyl-CoA hydratase/isomerase, catalyzes second step in fatty acid β-oxidation |  |  |
|  | MGG01180 | 3-hydroxyacyl-CoA dehydrogenase, catalyzes third step in fatty acid β-oxidation | I | 252 RLDGAARMPSKL 263 |
|  | MGG09512 | 3-ketoacyl-CoA thiolase, catalyzes final step in fatty acid β-oxidation | II | 3 ALDRLQQIGGQLSGA 17 |
|  | MGG13647 | 3-ketoacyl-CoA thiolase A, catalyzes final step in fatty acid β-oxidation |  |  |
|  | MGG10700 | β-ketoacyl-CoA thiolase, catalyzes final step in fatty acid β-oxidation | II | 2 AVERLGSILKHLTPG 16 |
|  | MGG09516 | α-methylacyl-CoA racemase, catabolism of branched-chain fatty acids | I | 392 DGALVLNDKSKL 403 |
|  | MGG05138 | 2,4-dienoyl-CoA reductase, β-Oxidation of unsaturated fatty acids (SPS19) | I | 302 SKNIKSGRKSKL 313 |
|  | MGG07309 | Delta(3,5)-Delta(2,4)-dienoyl-CoA isomerase, isomerization step in β-oxidation of unsaturated fatty acids | I | 274 GIQKRKATFAKL 285 |
|  | MGG00359 | Delta(3,5)-Delta(2,4)-dienoyl-CoA isomerase, isomerization step in β-oxidation of unsaturated fatty acids |  |  |
| Predicted genes involved in glyoxylic acid cycle | | | | |
|  | Name | Characters/functions | PTS | Related sequence |
|  | MGG04895 (*ICL1*) | Isocitrate lyase , key enzyme in glyoxylic acid cycle | II | 229 HINRLVAIRAQADIM 243 |
|  | MGG02616 | [Isocitrate lyase](http://www.broadinstitute.org/annotation/genome/magnaporthe_comparative/GeneFamilyTree.html?sp=S827486939) , key enzyme in glyoxylic acid cycle |  |  |
|  | MGG02813 | Malate synthase A, key enzyme in glyoxylic acid cycle | I | 531 TTPGSARPASKL 542 |

The open reading frames (ORF) encoding key enzymes involved in lipids degradation were first identified by searching the enzyme names against the *M. oryzae* genome database (<http://www.broadinstitute.org/annotation/genome/magnaporthe_comparative/MultiHome.html>), and then used as core sequences to re-search against the database using blastP procedure to get more potential candidates. The candidates were then checked in the NCBI database using blastP procedure to pick out the ORFs predicted to encode the enzymes. The PTS1 motifs were predicted by the PTS1 predictor (<http://mendel.imp.ac.at/mendeljsp/sat/pts1/PTS1predictor.jsp>) with the parameter FUNGI-specific function . The PTS2 motifs were predicted by searching the consensus (R/K)-(L/V/I)-X5-(H/Q)-(L/A) .
